# Protist impacts on marine cyanovirocell metabolism

**DOI:** 10.1038/s43705-022-00169-6

**Published:** 2022-10-01

**Authors:** Cristina Howard-Varona, Simon Roux, Benjamin P. Bowen, Leslie P. Silva, Rebecca Lau, Sarah M. Schwenck, Samuel Schwartz, Tanja Woyke, Trent Northen, Matthew B. Sullivan, Sheri A. Floge

**Affiliations:** 1 Department of Microbiology, The Ohio State University, Columbus, OH USA; 2 Lawrence Berkeley National Laboratory, Berkeley, CA USA; 3 Department of Ecology and Evolutionary Biology, University of Arizona, Tucson, AZ USA; 4 Department of Biology, Wake Forest University, Winston Salem, NC USA; 5 U.S. DOE Joint Genome Institute, Berkeley, CA USA; 6 Department of Civil, Environmental and Geodetic Engineering, and Center of Microbiome Science, The Ohio State University, Columbus, OH USA; 7 Present Address: U.S. DOE Joint Genome Institute, Berkeley, CA USA; 8 Present Address: Syft Technologies, Ltd, Christchurch, 8024 New Zealand; 9 Present Address: Department of Cellular and Molecular Medicine and Biomedical Sciences Graduate Program, University of California San Diego, La Jolla, CA USA; 10 Present Address: Scripps Institution of Oceanography, University of California, San Diego, La Jolla, CA USA; 11 Present Address: Microbial and Environmental Genomics, J. Craig Venter Institute, La Jolla, CA USA

**Keywords:** Bacteriophages, Water microbiology

## Abstract

The fate of oceanic carbon and nutrients depends on interactions between viruses, prokaryotes, and unicellular eukaryotes (protists) in a highly interconnected planktonic food web. To date, few controlled mechanistic studies of these interactions exist, and where they do, they are largely pairwise, focusing either on viral infection (i.e., virocells) or protist predation. Here we studied population-level responses of *Synechococcus* cyanobacterial virocells (i.e., cyanovirocells) to the protist *Oxyrrhis marina* using transcriptomics, endo- and exo-metabolomics, photosynthetic efficiency measurements, and microscopy. Protist presence had no measurable impact on *Synechococcus* transcripts or endometabolites. The cyanovirocells alone had a smaller intracellular transcriptional and metabolic response than cyanovirocells co-cultured with protists, displaying known patterns of virus-mediated metabolic reprogramming while releasing diverse exometabolites during infection. When protists were added, several exometabolites disappeared, suggesting microbial consumption. In addition, the intracellular cyanovirocell impact was largest, with 4.5- and 10-fold more host transcripts and endometabolites, respectively, responding to protists, especially those involved in resource and energy production. Physiologically, photosynthetic efficiency also increased, and together with the transcriptomics and metabolomics findings suggest that cyanovirocell metabolic demand is highest when protists are present. These data illustrate cyanovirocell responses to protist presence that are not yet considered when linking microbial physiology to global-scale biogeochemical processes.

## Introduction

Microbes drive planetary nutrient and energy cycles, but they do so under bottom-up (resources) and top-down (predation and infection) constraints within intricate food webs. In the oceans, resource availability was historically thought to affect microbial biomass and composition more strongly than predation [1]. However, recent global ocean species interaction networks suggest that biotic factors are stronger predictors of microbial community structure than abiotic factors [2]. Predation rates are commonly on par with microbial growth in low-nutrient surface ocean waters. However, the fate of nutrients differs by predator [3–6], with the paradigm being that viruses “shunt” carbon (C) and nutrients toward the dissolved phase [7–10] or “pump” C to the deep ocean [11], and heterotrophic protists serve as a critical link between lower and higher trophic levels [12–14]. Yet, viral and protistan interplay in controlling prokaryote populations and nutrient cycling [15–17] remains poorly characterized, despite indications of potential synergism of viral and protist impacts on prey populations [16, 18–20] and viral influence on protist predation and growth [21, 22].

In nature, *Synechococcus*, which together with *Prochlorococcus* contributes about one-quarter of total global oceanic primary productivity and is predicted to become more abundant with continued climate change [23], is both preyed upon by protists and infected by viruses (phages). For protists, field data show that they reduce *Synechococcus* abundances via direct ingestion (grazing) [24], while laboratory experiments demonstrate that protist predation alters freshwater *Synechococcus* cell morphology [25–27] in ways that could impact photosynthesis or nutrient cycling. For viruses, virus-infected cells (virocells) are physiologically and metabolically distinct from uninfected cells and display unique metabolite footprints that could impact the surrounding ecosystem [28–33]. For cyanobacteria virocells (cyanovirocells) specifically, while the physiological response of *Synechococcus* to infection has been characterized via genome-wide transcriptomics [34, 35] and photophysiology [36–38], the broader cellular response (e.g., metabolomics) and how the presence of a protist impacts cyanovirocell metabolic reprogramming and ecosystem footprints have not been studied. Ecosystems are comprised of communities of simultaneously interacting organisms, and yet phage-protist-cyanobacteria interactions remain understudied.

Here we assess phage-protist-cyanobacteria interactions by examining how population-level marine *Synechococcus* virocell physiology is impacted by the presence of a protist. We use a systems biology approach including time-resolved genome-wide transcriptomics, endo- and exo-metabolomics, photosynthetic efficiency measurements, and microscopy in a culture-based model system. Together these efforts provide mechanistic grounding to interactions among T4-like phages, *Synechococcus*, and dinoflagellates implicated in microbial interaction networks that are highly predictive of global ocean carbon flux [39].

## Materials and methods

### Culture conditions

Using semi-batch culturing, axenic *Synechococcus* strain WH8102 was grown in SN media prepared with sterile 0.1 μm-filtered, autoclaved seawater obtained from Scripps Pier in January 2014. Cultures were maintained at 20 ± 1 °C on a light: dark cycle of 14 h:10 h at 60 μmol photons m^−2^ s^−1^. The protist *Oxyrrhis marina* (CCMP3375; NCMA), a dinoflagellate of size 10–20 μm, was grown in Scripps Pier (January 2014) seawater with 1 × 10^5^ cells mL^−1^ *Isochrysis galbana* (CCMP1323; NCMA) as prey. Prey levels were depleted just prior to the experiment (see below).

The T4-like Myophage S-SSM5 (dsDNA genome of 176.18 Kbp) was propagated on axenic exponentially growing WH8102 within 14 days of beginning the combined phage infection and protist co-culture experiment, filtered through a 0.45-μm Supor filter (Pall Corp, cat. no. PN4614) and stored in the dark at 4 °C. Phage titer was determined by the most probable number assay [40, 41] using three independent dilution series on WH8102 grown in 96-well plates under the growth conditions described above. Preliminary viral infection dynamics were assessed using exponentially growing WH8102 at 60 μmol photons m^−2^ s^−1^ with a light: dark cycle of 14 h:10 h.

### Sampling

Twenty liters of exponentially growing *Synechococcus* strain WH8102 were aseptically pooled and split into four treatments: cyanobacteria only, cyanobacteria with phage, cyanobacteria with protist, and cyanobacteria with both phage and protist, with four biological replicates of each (each 1.2 L volume). Phage infection was initiated at the onset of the light cycle in eight separate flasks, each with 3 × 10^7^ pfu mL^−1^ (phage) incubated with 2.8 × 10^7^ host cells mL^−1^ (+phage treatment average) and 2.9 × 10^7^ host cells mL^−1^ (+phage and protist treatment average) for a multiplicity of infection of 1.1. Encounter theory estimates based on the infectious phage to host cell numbers present in the infection incubation predict that 67% of WH8102 cells would be phage-infected. After 1 h, cells were centrifuged and the supernatant containing free phages was removed, cells were washed and resuspended in spent culture medium (same for the cyanobacterium-only and cyanobacteria with protist treatments), and these samples were counted via flow cytometry. *Synechococcus sp*. starting concentrations were 1.9 ± 0.3 × 10^7^, 1.7 ± 0.1 × 10^7^, 2.2 ± 0.1 × 10^7^, and 1.7 ± 0.3 × 10^7^ cells mL^−1^ in the untreated control cyanobacteria only, phage infection, protist co-culture, and combined phage and protist treatments, respectively (Ave ± SD). In addition, two 1.2 L replicates of a 0.45-μm-filtered medium served as a control for exometabolite analyses.

Protist *Oxyrrhis marina* (CCMP3375) cells were allowed to reduce *Isochrysis sp*. CCMP1323 maintenance prey concentrations over 3 days. CCMP3375 was concentrated 1 h prior to the experiment via gentle gravity filtration over 10 μm pore size polyethersulfone membrane filters (PES, Sterlitech Corp. cat. no. PES 8025100), with continual flushing of sterile (0.22-μm-filtered and autoclaved) seawater. *O. marina* CCMP3375 was added to both cyanobacteria and cyanobacteria with phage treatments at a final concentration of 43 cells mL^−1^ and final protest: *Synechococcus* ratio of 1:512,000 and 1:395,000, respectively, which marked the start of the experiment. All treatments were maintained at 20 ± 1 °C under 60 μmol photons m^−2^ s^−1^.

Samples (transcriptomics, metabolomics, flow cytometry, microscopy, photosynthetic efficiency) were collected between 2 and 12 h every ~2 h during the daylight portion of the light: dark cycle, by gently mixing and pouring 100 mL into a sterile 250 mL polycarbonate flask. Cell and phage abundance samples were preserved with a final concentration of 0.1% EM-grade glutaraldehyde (Acros Organics, cat. no. 233280250), stored at 4 °C for 15–30 min, then flash-frozen in liquid nitrogen and stored at −80 °C until counted via flow cytometry (*Synechococcus* and phage) or fluorescence microscopy (*O. marina*). Active fluorescence samples were stored in darkened tubes for 15 min at 20 °C, then immediately analyzed (see below). For transcriptomics, ~2 × 10^8^ *Synechococcus* cells were centrifuged at 10,000 *g* for 10 min, flash-frozen in liquid nitrogen, and stored at −80 °C until RNA extraction. For exo-metabolomics, ~2 × 10^8^ *Synechococcus* cells were filtered onto 0.22-μm pore size PES filters (Sterlitech, cat. no PES0225100) and the filtrate was stored at −20 °C until analysis. For endo-metabolomics, the ~2 × 10^8^ *Synechococcus* cells retained on 0.22-μm pore size PES filters were washed twice with 5 mL of sterile phosphate-buffered saline, and filters were transferred to Eppendorf tubes, flash-frozen in liquid nitrogen, and stored at −80 °C until extraction. All downstream analyses were performed with a minimum of three biological replicates.

### Photosynthetic measurements

From at least three biological replicates, the maximum photosynthetic efficiency of photosystem II (PSII) expressed as *F*_*v*_/*F*_*m*_ was measured using the FastAct Fast Repetition Rate fluorometer (FRRf; Chelsea Technologies Group, Surrey, UK). The minimum (*F*_*o*_) and maximum (*F*_*m*_) fluoresence yields, *F*_*v*_/*F*_*m*_ were calculated as ((*F*_*m*_–*F*_*o*_)/*F*_*m*_) using cells that were dark-adapted at 20 °C for 15 min prior to analysis. Prior to experimental analyses, optimum excitation parameters were determined experimentally using cell cultures as recommended by the instrument manufacturer (Chelsea Technologies Ltd., FastPro Manual, pp. 33). Based on these data, the instrument was programmed to generate single turnover saturation using 100 excitation flashlets on a 2-μs pitch with a combination of excitation wavelengths (450, 530, and 624 nm) and included a relaxation phase of 40 flashlets on a 50-μs pitch. Acquisition sequences were repeated five times with 100–120 ms between sequences, as recommended. Cell-free controls (0.2-µm-filtered cultures; Pall Supor Acrodisk, Cat. No. 4612) were used to determine blank values.

### Statistics

Besides the method-specific statistics employed for transcriptomics and metabolomics, which are explained in their respective sub-sections, all instances where “significance” is mentioned in the results and discussion section refer to a two-tailed *t*-test with a *p* value <0.05.

### Transcriptomics

Transcriptomes were generated for six timepoints from each condition and RNA was extracted from frozen pellets using Qiagen RNEasy Mini Kit, total RNA was quantified and quality-assessed using the 2100 Expert Prokaryote Total RNA Pico kit and Agilent Bioanalyzer, rRNA sequences were depleted using an Illumina Ribo-Zero Plus rRNA Depletion Kit, libraries were created using the Illumina TruSeq Stranded Total RNA Kit, and sequenced on a HiSeq-2500 1 TB device, yielding paired-end 2 × 101 bp reads. Between 13 and 29 million reads were obtained per sample (median = 18.5 million reads). BBtools v35.84 following JGI’s default pipeline was used on raw reads to remove those containing two or more “N” bases with an average quality score across the read <10, with a length ≤51 bp, containing known Illumina artifacts, or mapping to PhiX, human, cat, dog, and mouse genomes with the identity of ≥93%. BBtools v35.84 trimmed reads to remove known Illumina artifacts in 5’ and 3’ ends, or when with a base quality score under 6 on 3’. Differential expression analyses were conducted with a custom-made R script used in numerous prior phage-host ‘omics studies [30, 42, 43]. Treatment samples were compared to their corresponding untreated control at each time point (e.g., T2 cyanovirocells versus T2 cyanobacteria only; T2 cyanovirocells with protist versus T2 cyanobacteria only).

### Metabolomics

For differential analyses, peak height was log_10_-transformed using a custom R script and used as a proxy for metabolite abundance. Metabolites detected (raw peak height ≥10,000) in <2 biological replicates were excluded. Differential abundance between cyanobacteria only (control) and each infection and/or protist co-culture treatment was computed using a fold change (FC) between log_10_-transformed peak heights (averaged across replicates). False discovery rate (FDR) was estimated using a *t*-test with Benjamini–Hochberg-corrected *p* values. A metabolite was considered significantly different between control and treatment if log_10_FC ≥ 0.1 (an approximate change of 25% between treatment and control) and FDR ≤ 0.05 (5% false positives).

## Results and discussion

### Experimental design and infection dynamics

**Fig. 1:**
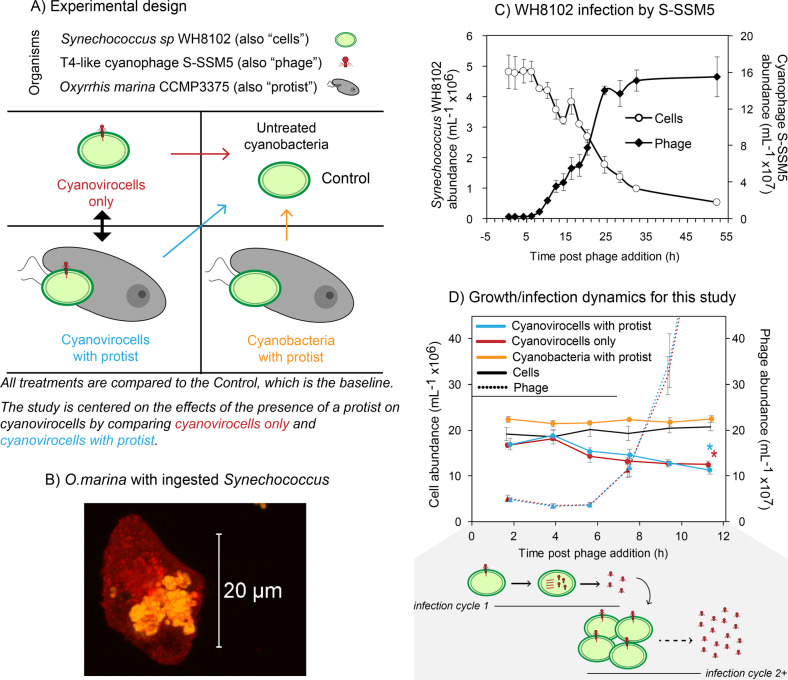
Experimental design and impacts of a protist on cyanobacteria and cyanobacteria virocells (cyanovirocells). **A** Experimental design. *Synechococcus* WH8102 cells were infected with the T4-like myophage S-SSM5 to generate cyanovirocells. Cyanovirocells were studied over the course of infection either alone (“Cyanovirocells only” treatment) or in the presence of the protist *Oxyrrhis marina* (“Cyanovirocells with protist”). Arrows denote the comparisons: all treatments are compared against the Control to detect significant changes to the transcripts, metabolites, and photosynthetic efficiency. In addition, the focus of this study is the cyanovirocell responses to the presence of a protist, which is the contrast between cyanovirocells only and cyanovirocells with protist. **B** Fluorescent microscopic image of *O. marina* with ingested *Synechococcus* cells (protists were stained red with WGA - Alexa 488 and *Synechococcus* were detected with phycoerythrin pigment autofluorescence). Image was acquired using Zeiss LSM 710 confocal microscope with a 63× (1.4 N.A.) objective. **C** General infection dynamics of *Synechococcus* infected with S-SSM5, followed over 55 h post phage addition. This time course captures multiple cycles of infection. **D** Cell and phage abundances over the course of this study. Samples for genome-wide transcriptomics, endo-metabolomics, exo-metabolomics, and microscopy and photosynthetic efficiency measurements were taken every 2 h between 2 and 12 h post phage and/or protist addition. This time course captures a population-level view of infection, whereby more than one infection cycle is taking place (shaded in gray): the phages released from “infection cycle 1” infect new cells and undergo “infection cycle 2+” (i.e., two or more asynchronized infection cycles may be happening at this time). Both the burst size (here depicted as 4 phages released per infected cell), and the number of infection cycles are simplified. Cell abundance decreases significantly from start to end of ‘omics sampling during phage infection (*t*-test, *p* value <0.05), with or without protist (indicated by an asterisk for each treatment), and it does not significantly change in the presence of the protist alone. All experiments included a minimum of three biological replicates and the average with standard error is shown.

We sought to understand the effect that the presence of a protist, regardless of significant predation, would have on a population of phage-infected marine *Synechococcus* (i.e., virocells), through a time-resolved systems biology approach. To this end, we generated *Synechococcus* virocells (i.e., cyanovirocells) by adding the T4-like Myovirus S-SSM5, and we compared these cyanovirocells to cyanovirocells co-cultured with the protist *O. marina*. These cyanovirocells were compared to uninfected *Synechococcus* co-cultured with the protist, and the baseline for all treatments was a *Synechococcus* control lacking both phage and protist (Fig. 1A). Microscopy measurements confirmed that approximately half the protist population were actively feeding upon *Synechococcus* (Fig. 1B), even though they did not significantly alter their overall abundance due to the low protist: prey ratio. Furthermore, we calculated an encounter rate of 444 prey cells mL^−1^ s^−1^ following conservative approaches [44, 45], which represents 8–97% of the total prey population directly encountered by the protist by 2 and 10 h, respectively. *O. marina* contacting prey would presumably induce prey stress signals, as previously found [46, 47], but neither direct prey ingestion nor prey contact is necessary for the protist to elicit prey responses [48–52]. Here we investigate *Synechococcus* virocell physiological responses to the presence of the protist, rather than responses to predation.

**Fig. 2:**
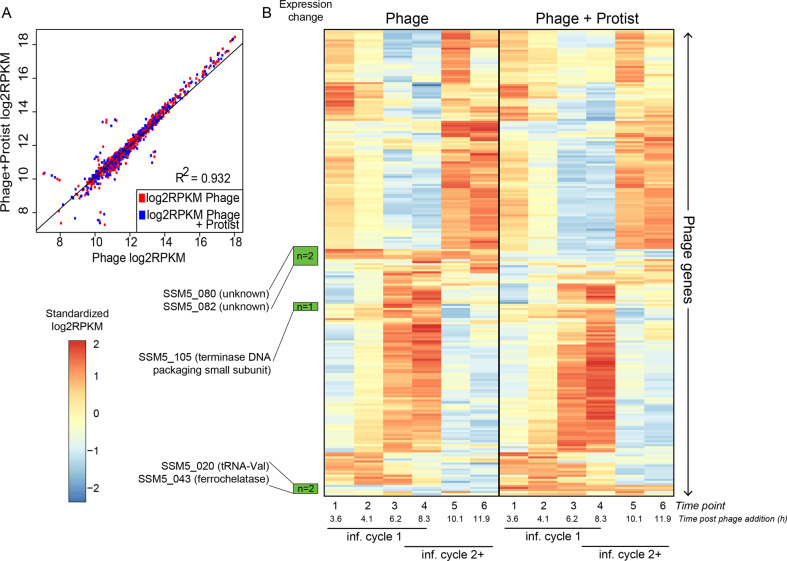
Phage expression in the presence and absence of the protist. **A** Pearson correlation of the phage gene expression values (as log_2_RPKM) with and without protist shows that phage expression levels are largely the same in both treatments. **B** Heatmap representing the expression (as standardized log_2_RPKM) of all phage genes (vertical) in the treatment with (right) or without (left) protists. Genes that change temporal dynamics in the presence of protists are marked in green (5 in total) and their annotations are displayed. The known transcriptional categories for this and generally the T4-like myophages (early, middle and late-expressed genes) are unaltered with protist for all except 5 genes (see Supplementary text). The time course for the infection captures more than one infection cycle: the first one with the phages added at the start of the experiment (“inf. cycle 1”) and the ones that follow once the phages are released from “inf. cycle 1” and infect new cells (“inf. cycle 2+”).

As for the phage, we examined infection dynamics over the course of multiple infection cycles, expecting the first infection cycle to be similar to that of the closely related T4-like phage Syn9 (77% average nucleotide identity; Supplementary Fig. S1) infecting our same host [34, 53]. Results indicated that, indeed, the phage had a ~6–8-h latent period and significantly reduced *Synechococcus* population abundances (Fig. 1C). These dynamics were maintained when the protist was added (Fig. 1C), confirming that protist addition did not catastrophically interfere with phage infection. Similarly, phage infection did not alter protist feeding upon *Synechococcus* (Supplementary text). Finally, phage transcription was also mostly unaltered when the protist was added (Fig. 2), as the phage maintained the temporal transcriptional dynamics expected for T4-like myoviruses [34, 54] with or without protist, and only five phage genes significantly changed expression with the protist (Supplementary Figs. S2 and S3 and Supplementary text). Overall, protists were unaffected by phages and phage infection was unaffected by protists. The experimental design thus enables us to examine the ways in which cyanovirocells respond to external protist presence, not necessarily predation, from a transcriptional, metabolic, and ecological perspective.

### Protist impact on cyanovirocell endometabolites

To date, virocell studies have demonstrated that while phage transcription may largely be invariant, the host has a unique transcriptional response depending on the phage and the environment [34, 42, 43, 55, 56]. However, the transcriptional and metabolic responses of virocells during co-culture with protists are unknown.

**Fig. 3:**
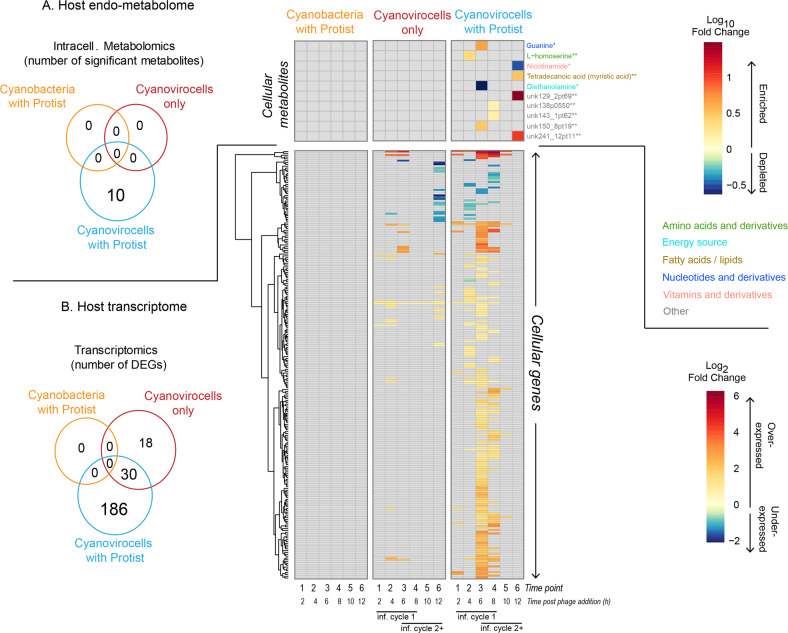
Host genes and metabolites altered in each treatment relative to the untreated control. **A** Number of intracellular metabolites detected as significantly changing in each of the treatments (Venn diagram) and their log_10_FC value in the treatment relative to the untreated cells (heatmap). Compound names are colored by the class to which they belong, and the confidence in their detection is indicated by an asterisk next to the name. Confidence of level 1 (1 asterisk) indicates the compound had three identification criteria in positive or negative mode (i.e., retention peak, mz, and msms), whereas level 2 (2 asterisks) denotes two of the three criteria were met. Compounds denoted as “Other” represent detected features that were not identified. **B** Number of host genes differentially expressed in response to protist only (“cyanobacteria with protist”), phage only (“cyanovirocells only”), or phage plus protist (“cyanovirocells with protist”) pictured in a Venn diagram as well as their heatmap, which denotes gene expression as log_2_fold change (FC) values in the treatment compared to the untreated control over the experiment time course. Gray heatmap cells denote genes or metabolites that did not significantly change their expression or abundance, respectively, in the treatment relative to the control. All represented data derive from the average of a minimum of three biological replicates in which the treatment was compared to the uninfected control (cells only, no phage, no protist). The time course for the cyanovirocells and cyanovirocells with protist captures more than one infection cycle: the first one with the phages added at the start of the experiment (“inf. cycle 1”) and the ones that follow once the phages are released from “inf. cycle 1” and infect new cells (“inf. cycle 2+”).

We investigated whether the presence of a protist would alter cyanovirocell transcriptional and metabolic profiles given the stress of protist–prey contact, handling, and ingestion/rejection [46–52, 57, 58]. We first investigated population-level intracellular metabolite profiles, which has not been explored previously. For this, we compared each treatment (cyanobacteria with protist, cyanovirocells without protist and cyanovirocells with protists) to the control (cyanobacteria only) lacking both phages and protists. We found that 0, 0, and 10 intracellular metabolites changed in each of those treatments relative to the control, respectively (Fig. 3A and Supplementary Table S1). The ten metabolites significantly changing in the cyanovirocells with protists are fewer than those reported in the three other prior temporally resolved virocell endometabolomic studies [59–61]. A detailed comparison with those studies (Supplementary Table S2) revealed that we used more stringent criteria for significance and that, had we used the prior, more permissive analytical procedures, those would have identified up to 17× more metabolites (Supplementary Table S3 and Supplementary text). As marine endo- and exo-metabolomics analyses are not yet standardized across the field, we opted for a more conservative approach for our metabolomics analyses.

Of the ten endometabolites significantly changing in the cyanovirocells with protist, the majority (60%, *n* = 6) changed in the population undergoing the first infection cycle (ending at 6–8 h), whereas the rest changed at 12 h post phage addition; in the population undergoing subsequent infections (Fig. 3A). Of the six metabolites changing within the first infection cycle, five were enriched (guanine, L-homoserine and three others with no database hits, thus termed “unknown”) and one was depleted (diethanolamine) in the protist-treated cyanovirocells relative to the control lacking phages or protists, and all between 4 and 8 h. Metabolites could be enriched because cyanovirocells either increase production intracellularly or incorporate them from the environment. Both guanine (a nucleotide) and L-homoserine (an amino acid) are building blocks that could be resources for phage reproduction, as it has previously been posited from transcriptome [55, 60, 62–65] and metabolome [59–61] studies of other phage infections. Especially in cyanobacteria, cyanophages are thought to be highly proficient at deviating cellular resources toward phage replication [31, 34, 66], which in this case would fuel the middle and late stages of infection. Alternatively, homoserine is also known to increase in cells responding to stress, likely as a consequence of protein degradation [67]. Therefore, the enrichment of these metabolites in the protist-treated cyanovirocells could reflect both stress and a need for additional resources for phage replication that are not as needed in the protist-free cyanovirocells.

Finally, the remaining four endometabolites that changed after the first infection cycle included three enriched (the lipid myristic acid and two more “unknown”) and one depleted (nicotinamide, a vitamin B3 derivative) compound. Here the signal derives from an asynchronous population composed of an uninfected minority, as well as a majority of cells undergoing different stages of infection. The lipid increase is consistent with viral infection [59, 68–70] and cyanophage-mediated restructuring of cellular membranes [71], as well as with microbial responses to protists (without phages) [72]. The decrease in intracellular nicotinamide may reflect cyanovirocells utilizing it for survival, given its critical biological role in myriad enzymatic reactions [73].

### Protist impact on cyanovirocell transcription

Focusing next on gene expression, we again compared all treatments (cyanobacteria with protist, cyanovirocells only, and cyanovirocells with protist) to the untreated control. Each of those had 0, 48, and 216 differentially expressed (DE) genes (Fig. 3B and Supplementary Table S1). The 48 DE genes in the cyanovirocells only treatment represent a smaller host response than that reported for this same host during infection by myovirus Syn9 [34] which we attribute to differences in both the phage used and the experimental design as explained in the Supplementary text. The lack of DE genes in the protist-only treatment suggests that the transcriptional differences across the two cyanovirocell treatments (the 216 vs 48 DE genes) were due to protist presence, with a 4.5-fold increase in the number of host genes changing expression in the cyanovirocells in response to the protist. Both the direction (increased or decreased expression relative to the control; here “over-expressed” and “under-expressed”, respectively) and timing of cyanovirocell gene expression also varied with and without protist, as follows. Host genes were mostly over-expressed—65 and 89% in the absence and presence of the protist, respectively (Supplementary Fig. S5A)—thus suggesting that a larger number of host genes enhanced their expression in the cyanovirocells co-cultured with the protist than without it. In addition, when protist was added, the cellular transcriptional response was greater toward the end of the infection cycle, as the majority (52%, *n* = 153) of the genes were DE at 6 h post phage addition, compared with only 18% at that same time without the protist (Supplementary Fig. S5B). Thus, when protist was added, host transcriptional responses were greatest toward the end of the first infection cycle. Again, the majority (97%, *n* = 149) of those 153 genes were over-expressed.

We next examined the functions of these 153 host transcripts that responded at 6 h in the cyanovirocells with protists by grouping them into functional categories. The category with the most genes was the “unknown” (not functionally annotated, *n* = 74 genes) and its average fold change (aFC) of expression in the cyanovirocells treated with the protist relative to the cyanobacteria only control was 3.7 (Supplementary Fig. S5C), which highlights the need for further experimental work to characterize unknown genes even in relatively well-studied marine cyanobacteria.

**Fig. 4:**
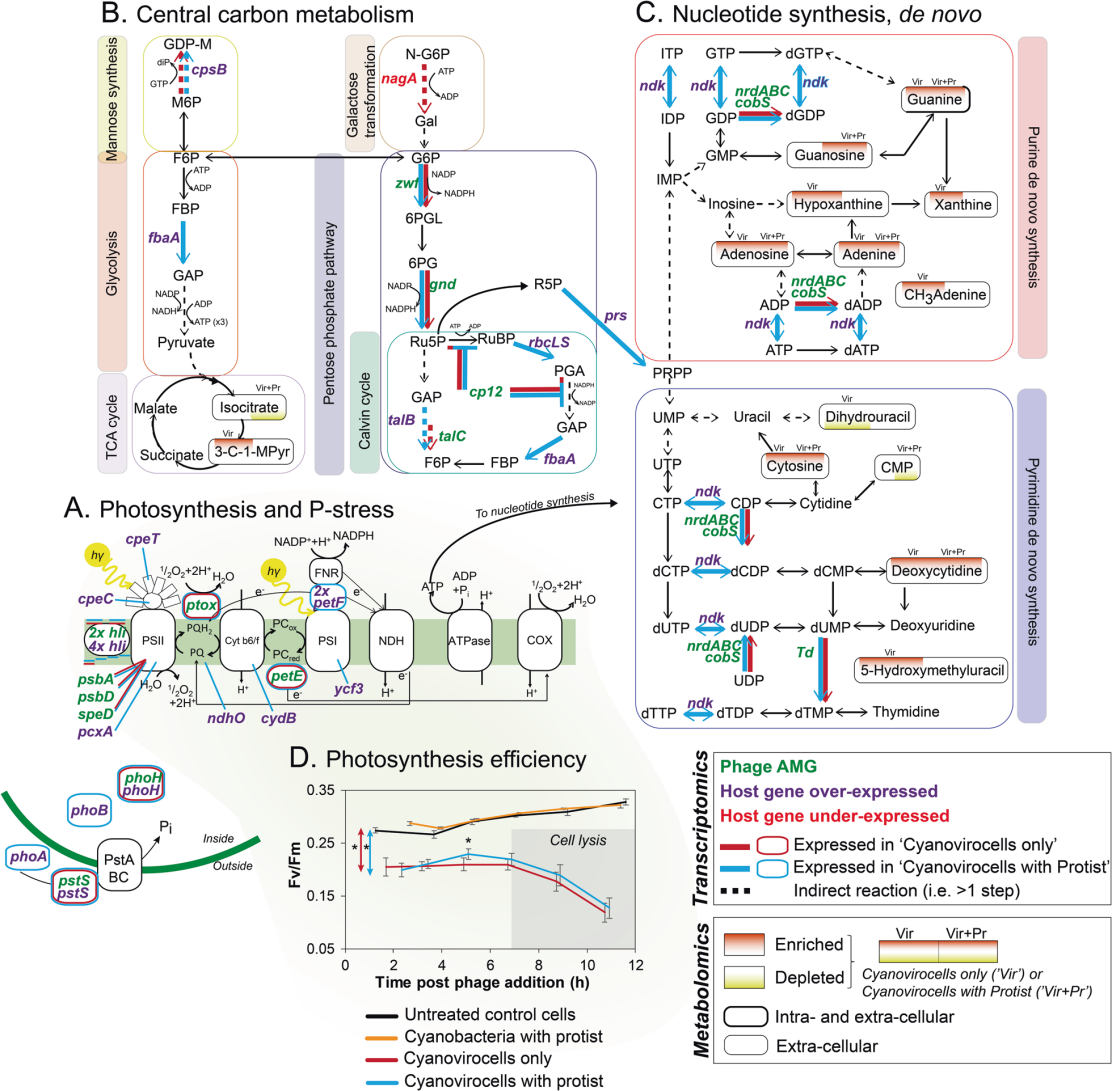
Cyanovirocell energy and resource metabolic pathways responding to the protist. **A** Photosynthesis and phosphate (P) stress. **B** Central C metabolism, including mannose synthesis, glycolysis, galactose metabolism pentose phosphate pathway (PPP), Calvin cycle, and the Tricarboxylic acid (TCA) cycle. **C** De novo purine and pyrimidine nucleotide synthesis. Phage auxiliary metabolic genes (AMGs) are in green text. Host over-expressed genes are in purple, while the under-expressed one is in red. The color of the arrow or the border (orange or blue) denotes if the gene is expressed in response to phage only or phage and protist. Metabolites that change significantly have a background shade colored red (if enriched) or yellow (if depleted) and the position of the shade denotes under which treatment they are altered: in the cyanovirocells only or in the cyanovirocells co-cultured with protists. **D** Photosynthetic efficiency of *Synechococcus* cells under each condition. Asterisks indicate when treatments are significantly different (*t*-test, *p* value <0.05). Cyanovirocells and cyanovirocells with protist both have significantly lower photosynthetic efficiency than uninfected cells (red and blue arrows with asterisks, respectively), but cells with just the protist do not (*t*-test, *p* value >0.05). Cyanovirocells with protist significantly increase in photosynthetic efficiency from ~2 to ~5 h post infection relative to cyanovirocells alone (*t*-test, *p* value <0.05). Abbreviations can be found in the Supplementary text.

The three most highly expressed categories with known functions were “Phosphate Metabolism” (aFC = 5.8, *n* = 3 genes), “Central Carbon Metabolism” (aFC = 5.4, *n* = 5 genes) and “Protein Metabolism” (aFC = 4.9, *n* = 15 genes) (Supplementary Fig. S5C). The “Phosphate Metabolism” category included the inorganic phosphate (Pi) transporter *pstS* and the transcriptional regulator *phoB* that respectively transport Pi into the cell and activate downstream cellular metabolic pathways under P-limitation [74]. Expression of these genes suggests that the cyanovirocells were P-limited, which is expected from cyanobacteria-cyanophage gene expression studies lacking protists [34, 75–78]. As *phoB* was not over-expressed without the protist, only *pstS* (Fig. 4A), assuming the PhoB protein is also highly produced, these data suggest that the presence of the protist induces a low-P-mediated cell-wide metabolic change in the cyanovirocells.

Next, the “Central Carbon Metabolism” category included genes involved in glycolysis, pentose phosphate pathway (PPP), and the Calvin cycle. These genes have been found to be expressed during cyanophage infection [34, 78, 79] and are assumed to shift cellular energy production toward phage replication. Given that they were only over-expressed in the protist-treated cyanovirocells, it suggests that protist presence changes the energy needs of the cyanovirocells, especially toward the end of infection (see below).

Finally, the “Protein Metabolism” category mainly included genes for chaperones such as Hsp20, GroES, and GroEL, which were not DE in the cyanovirocells when the protist was absent. These chaperones were found over-expressed in *Polynucleobacter asymbioticus* responding to a protist [47], and are known to respond to various stresses in cyanobacteria, including phage infection [80], heat-shock, salinity, and oxidative stress [81, 82], but can also protect the photosystem II from photoinhibition [81], or help assemble key proteins, including Rubisco [83]. Since the host Rubisco genes as well as phage structural and lysis genes (Supplementary Fig. S2) were also over-expressed at this time, these host chaperones may help assemble key host and phage proteins during the final stages of infection.

We next asked if the presence of protists enhanced cellular stress. While only one gene in the “Stress” category was DE (and under-expressed, relative to the control) in the cyanovirocells without protist, 14 genes were DE (and over-expressed, relative to the control) throughout the infection when the protist was added, with the majority (*n* = 8 genes) over-expressed with an aFC = 3.4 at 6 h post phage addition, including the above chaperones (Hsp20, GroES, and GroEL), which contribute to stress as mentioned above, and Rubisco (Supplementary Fig. S5D). These data provide further evidence that the stress was higher in the cyanovirocells when the protist was present than when it was absent. We posit this stress could increase the energy and metabolic demand of phage infection in the protist-exposed cyanovirocells, even without significant predation. This could happen either directly through metabolic changes [84, 85], or indirectly through enhancing antipredator strategies that are known to occur in aquatic bacteria (e.g., increased motility, metabolite release), which are energetically costly [3, 86, 87].

Altogether, the transcriptomics and endo-metabolomics data suggest that the presence of the protist, even without significant predation, (i) augments the transcriptional and endometabolomic response of cyanovirocells, (ii) shifts cyanovirocell intracellular physiological responses largely toward the end of an infection cycle, and (iii) induces a larger stress, energy and resource demand in the cyanovirocells than in its absence.

### Protist impact on cyanovirocell resource and energy production

The above results suggested that cyanovirocells nearing the end of the infection cycle required more cellular energy and resources in the presence of protists than in their absence. Cyanophages often encode auxiliary metabolic genes (AMGs) that alter (a) host photosynthesis and central C metabolism pathways (e.g., PPP and Calvin cycle) for obtaining energy (ATP), reducing power (NADPH), and nucleotide precursors (ribose-5-phosphate), and (b) P acquisition and nucleotide synthesis pathways for deviating cellular metabolism toward building new phages [33–37, 66, 77–79, 88–94]. Specifically, photosynthetic reaction center proteins, proteins that stabilize such reaction centers, and proteins that enhance light harvesting during infection are among the cyanophage AMGs that “boost” host machinery to ensure photosynthesis during infection. In addition, AMGs from central C metabolism pathways inhibit the Calvin cycle to prevent ATP consumption and to redirect C flow toward the PPP, from which NADPH and ribose-5-phosphate are obtained. With these resources, as well as with acquired P (with P-stress transporters), cyanophages can replicate their genomes (reviewed in [31, 78]; Fig. 4A–C).

We next examined the expression of the phage AMGs and host genes involved in those metabolic pathways. Phage S-SSM5 encodes AMGs involved in all of these pathways: (i) P-stress (*pstS, phoH*), (ii) photosynthesis (*petE, ptox, hli* (*n* = 2), *psbA*, *psbD, speD*), (iii) central C metabolism (*zwf, gnd, talC, cp12*), and (iv) nucleotide synthesis (*nrdA*, *nrdB, nrdC, td, cobS*) (Fig. 4A–C) [31, 32, 78], and were all expressed regardless of protist presence (Supplementary Table S4 and Supplementary Fig. S6). Contrastingly, the cyanovirocells over-expressed 4 and 23 (the same 4 plus 19 more) host genes in the absence and presence of the protist, respectively, relative to the control (Supplementary Table S5 and Supplementary Fig. S7). These genes are for (i) P-stress (*phoA, phoB, pstS* and *phoH*), (ii) photosynthesis (*hli, cpeT, pcxA, cpeC, ndhO, cydB, petF, ycf3*), (iii) central C metabolism (*cpsB, fbaA, talB, rbcL and rbcS*), and (iv) de novo nucleotide synthesis (*prs* and *ndk*). These were involved in different steps of the reprogrammed metabolic pathways than the phage AMGs (Fig. 4), with transaldolase being the exception, as both the phage (*talC*) and bacterial (*talB*) homologs were expressed in the same step of PPP. Presumably, this is because an enzymatic “double dose” is helpful for that step, given that it may be a bottleneck of PPP [79], whereas the rest of the steps can be driven by just the phage or the host enzyme. Finally, most genes were expressed at the end of the infection (6–8 h, *n* = 21 genes) in the protist-treated cyanovirocells; Supplementary Fig. S7). These data suggest that the phage specifically directed over-expression of host genes complementary to its AMGs to boost metabolic pathways necessary for the last stages of phage reproduction with and without the protist, but more so in the presence of the protist given the larger number of DE genes.

In summary, protist-treated cyanovirocells had a greater number of over-expressed host genes involved in (a) P-stress, which suggests that cyanovirocells co-cultured with protists have higher P demands than cyanovirocells without protists (b) nucleotide synthesis, which comes into play after recycling host DNA [95] and is an energetically costly process, and (c) photosynthesis, which suggest a larger energetic demand on *Synechococcus* during phage infection under protist predation (Fig. 4A–C). Overall, these data suggest that cyanovirocells have higher resource and energy demand when exposed to protists, regardless of significant predation.

Given these findings, we sought to assess whether the protist imposed a larger energetic demand on cyanovirocells through measuring the photosynthetic efficiency *F*_*v*_/*F*_*m*_. While the overall initial *F*_*v*_/*F*_*m*_ of the *Synechococcus* cells were lower (0.20–0.29) than those observed in other photosynthesis studies of *Synechococcus* phage infection (0.6–0.7; [36–38]), the discrepancy is likely due the differences in irradiance and the bacterial strain used. Specifically, our cells were grown at 20 °C and 60 μmol photons m^−2^s^−1^ temperature and irradiance, respectively, and under these same conditions, three *Synechococcus* strains displayed *F*_*v*_/*F*_*m*_ values of 0.26–0.34 [96]. In addition, work with our same strain grown at 10 and 150 μmol photons m^−2^s^−1^ obtained *F*_*v*_/*F*_*m*_ values of 0.36 and 0.15, respectively [97].

The photosynthetic efficiency of cyanobacteria cells co-cultured with protists (*F*_*v*_/*F*_*m*_ values of 0.28–0.32) was not significantly different from control cells (*F*_*v*_/*F*_*m*_ values of 0.27–0.33) at any time point. This may be due to the low abundance of protists relative to the cyanobacteria. In contrast, the cyanovirocells, with or without protists, displayed significantly lower *Synechococcus* photosynthetic efficiency compared to the untreated control—by 35% as early as 2 h post-infection, and 64% by 12 h post-infection (Fig. 4D and Supplementary Fig. S8), which coincides with the first and subsequent infection cycles, respectively. The reduction in *Synechococcus* photosynthetic efficiency in response to phage infection is similar to that observed in virus-infected eukaryotic phytoplankton [98, 99], but contrasts with prior findings in *Synechococcus* or *Prochlorococcus* phage infection experiments [36, 37, 100], which demonstrated no change in *F*_*v*_/*F*_*m*_ prior to host cell lysis. This discrepancy is likely due to instrumental differences as we used a single instead of multiple turnover flash fluorometer, and this methodological difference enabled us to observe phage-mediated plastoquinol quenching, indicative of deviation of light energy away from C fixation toward ATP production, which is not detectable using multiple turnover flash fluorometers [101–103]. Consistent with this physiological measurement, phage plastoquinol terminal oxidase (*ptox*) was expressed during early infection with or without protist (Supplementary Fig. S6A), and *ptox* has been hypothesized to facilitate alternative electron flows that prevent damage to PSII [89, 104].

Finally, while cyanovirocell photosynthetic efficiency was overall lower than the cyanobacteria only control as mentioned above, protist-treated cyanovirocells had a significant increase of ~15% (±5%) just before the first infection cycle cell lysis (~5 h post-infection), relative to the protist-free cyanovirocells (Fig. 4D and Supplementary Fig. S8). These results show that the energy required for phage infection was higher with protist presence toward the end of infection. Together with the genome-wide transcriptomics and endo-metabolomics findings, these results suggest that cyanovirocells co-cultured with a protist require greater resources (reflected in the transcriptional and metabolic changes) and energy (reflected in increased photosynthetic efficiency and metabolic reprogramming) to support phage reproduction, regardless of whether protists were actively feeding upon *Synechococ*cus.

### Protist impacts on cyanovirocell extracellular metabolites

**Fig. 5:**
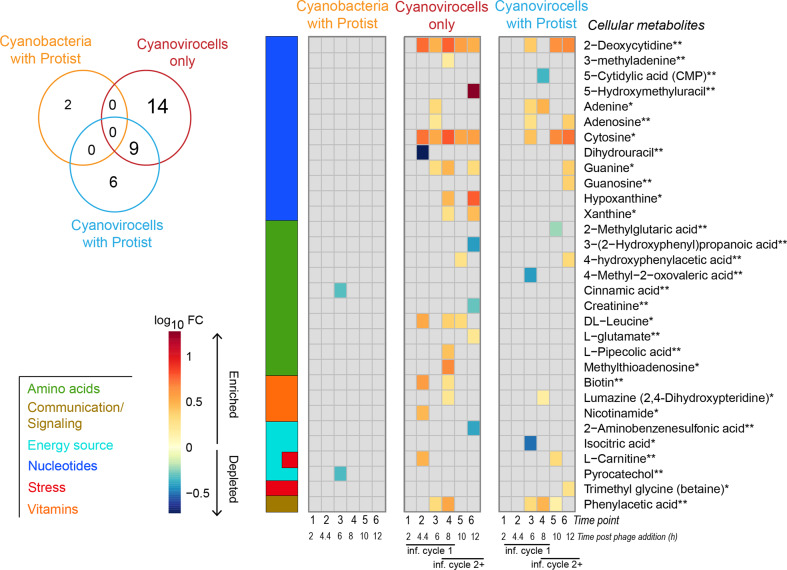
Changes in the extracellular metabolome of cyanobacteria across three treatments. Venn diagram: number of significantly changing metabolites detected extracellularly in each treatment. Heatmap: abundance of all significantly changing metabolites detected extracellularly with each treatment, in log_10_-fold change (FC) of the values in the treatment relative to the uninfected control. The colored bar on the left represents the class to which the metabolites belong. The confidence in metabolite detection is indicated by an asterisk next to the name. Confidence of level 1 (1 asterisk) indicates that the compound met all three identification criteria in positive or negative mode (i.e., retention peak, mz, and msms), whereas level 2 (2 asterisks) denotes two of the three criteria were met. The time course for cyanovirocells and cyanovirocells with protist captures more than one infection cycle: the first one with the phages added at the start of the experiment (“inf. cycle 1”) and the ones that follow once the phages are released from “inf. cycle 1” and infect new cells (“inf. cycle 2+”).

We next profiled the exometabolome of all treatments (cyanobacteria with protist, cyanovirocells only, and cyanovirocells with protist) for dissolved chemical cues relative to the untreated control [6, 86, 87], to evaluate whether the presence of a protist changes ecosystem footprints. A total of 2, 23, and 15 exometabolites significantly changed in each respective treatment (Fig. 5 and Supplementary Table S1). In the protist-only treatment, the two metabolites were cinnamic acid and pyrocatechol, which were significantly depleted. Their depletion could mean either that the protist induced *Synechococcus* to release fewer compounds, or that one of the organisms, the protist or *Synechococcus*, were utilizing these metabolites, thus leading to decreasing their extracellular abundance. As these two exometabolites were absent from the cyanovirocell samples, we interpret that the protist alone had a unique impact on *Synechococcus*’s exometabolome in that it was different from the impact induced by viral infection. We next focused on the exometabolite changes induced by viral infection with and without the presence of the protist.

In the cyanovirocells only treatment, we evaluated the timing and functional annotation of the 23 exometabolites that changed relative to the cyanobacteria only control (Fig. 5). Within the first 6 h of infection, 10 metabolites were enriched extracellularly, indicating pre-lysis phage-induced metabolite release from intact cells. Functional annotation suggested that the exometabolites were nucleotides (*n* = 5), amino acids (*n* = 1), vitamins (*n* = 2) and potential communication (*n* = 1) and stress (*n* = 1) molecules. These could be resources for cellular or phage growth (e.g., the nucleotides, amino acids, and vitamins [59, 61, 105]), or cell-to-cell communication molecules [106]. Such virus-induced changes could have consequences for the microbial food web. For example, viruses can induce biochemical changes in the coccolithophore *Emiliania huxleyi* that decrease copepod and protist grazing [22, 107]. These findings suggest that phages shape both the intra- and the extracellular landscape, and that cyanovirocells release nutrients prior to cell lysis. Alternatively, as this treatment was composed of ~67% cyanovirocells and ~33% uninfected cells (Supplementary Dataset), it is possible that the enriched exometabolites derive from uninfected cells responding to cyanovirocells. However, considering that the majority of cells were infected and phage-mediated metabolite restructuring and release has been previously reported [59, 61, 108, 109], we favor the hypothesis that the cyanovirocells are the exometabolite source. Regardless, the organic matter release undoubtedly would benefit the larger microbial community by providing resources [110].

The eight unique exometabolites that were enriched in the subsequent infection cycles (≥8 h) of the protist-free cyanovirocells were our next focus. We reasoned that these could derive either from a minority of cyanovirocells undergoing different infection stages or a majority of lysed cells. Given that they were not detected in prior timepoints, during the infection cycle, and before lysis, we presume that the latter scenario is most likely. These were amino acids (*n* = 4), nucleotides (*n* = 3) and vitamins (*n* = 1), thus supporting prior findings that viral lysis releases organic matter [59, 109, 111]. Together, the exometabolite data from the protist-free cyanovirocell treatment suggests that (i) stress and communication molecules were unique to intact cells, as they were found only in the first infection cycle, and (ii) cyanovirocell-derived amino acids, nucleotides, and vitamins are organic resources available to a microbial community pre- and post-cell lysis.

Finally, we evaluated the 15 exometabolites differentially detected in the cyanovirocells co-cultured with protists relative to the cyanobacteria only control, 9 of which were shared with the cyanovirocells only treatment (and were also enriched), and 6 of which were new (2 enriched, 4 depleted). The decrease in the number of enriched exometabolites in the cyanovirocells with protist treatment relative to the cyanovirocells only treatment suggests that either in response to the protist the cyanovirocells were decreasing export/diffusion, or that one of the microbes (cyanobacterial cells, cyanovirocells, or protists) were utilizing the metabolites. For example, *Sulfitobacter* virocells [59] have both been shown to uptake exometabolites, and both *Synechococcus* and the protist are capable of directly importing dissolved molecules [110, 112]. The 4 exometabolites were derivatives of a nucleotide, two amino acids, and a TCA cycle metabolite intermediate from which bacteria obtain energy. All but one were depleted during the first infection cycle (Fig. 5), suggesting consumption by the cyanovirocells to supply extra resources for phage infection in the presence of the protist.

### Ecological implications of protist-cyanovirocell interactions

**Fig. 6:**
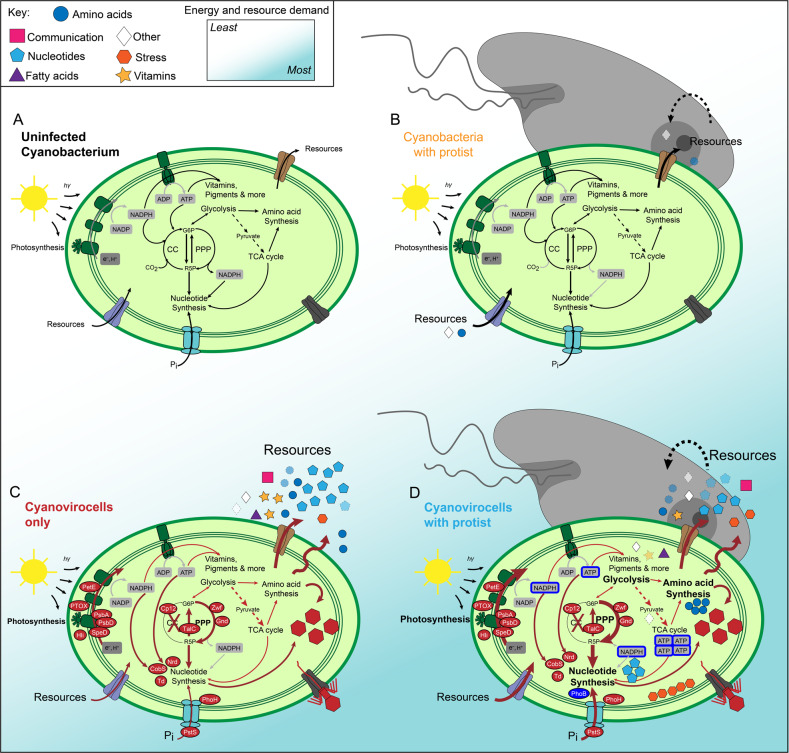
Metabolic reprogramming and ecosystem impact of protist- and/or phage-treated *Synechococcus*. **A** Uninfected cyanobacterium. **B** Cyanobacteria with protist. The presence of the protist alters the abundance of some exometabolites. **C** Cyanovirocells only. Cyanophages reprogram P-acquisition, photosynthesis, energy pathways, and nucleotide metabolism toward building new phages. **D** Cyanovirocells with protist. The energy (e.g., ATP) and resource (e.g., reducing power, phosphate, nucleotides, and amino acids) demand of phage infection is highest for the cyanovirocells co-cultured with a protist. Cyanovirocells have the largest changes in the exometabolites as seen from the release of nutrients, either via diffusion or active transport across the membrane (**C**, **D**). This nutrient pool is available for the ecosystem, including uptake by protists (**D**).

In this work we sought to move beyond the focus on the biogeochemical impacts of viral lysis and metabolic reprogramming of viral-infected cyanobacteria [31] toward building a conceptual model for the impacts of how protist presence impacts cyanovirocell infection dynamics and physiology (Fig. 6). To this end, we assessed cross-kingdom interactions between viruses, protists and cyanobacteria from the cyanovirocell perspective and its intra- and extracellular responses to the presence of a protist, with the untreated cell (Fig. 6A) as a baseline for multi-omics analyses. First, protist presence alone induced no measurable impact on *Synechococcus* endometabolites or transcripts, which could be due to the low relative abundance of the protist. However, protist presence did alter metabolite output, thus suggesting even low protist numbers have a measurable ecosystem impact (Fig. 6B).

Second, without the protist, *Synechococcus* had a small intracellular response to phage infection, with (i) very few transcriptional changes, (ii) expected phage AMG-driven metabolic reprogramming (reviewed in [31, 78]), and (iii) no significant impacts on detected endometabolites (Fig. 6C). Such a small intracellular impact suggests that this phage can replicate in this host using existing cellular resources, as previously observed for heterotroph-infecting phages that are well adapted to their host [30]. However, the phage-mediated cyanovirocell extracellular footprint was larger than the intracellular one, with multiple enriched exometabolites that spanned various nutrient classes (e.g., nucleotides, amino acids, vitamins, and communication/stress molecules) and which reinforce that cyanovirocells, similar to other known, marine organisms [113], can be a source of diverse “public goods” to feed and communicate with other organisms.

Third, when cyanovirocells were co-cultured with protists (Fig. 6D), even without significant population reduction from grazing, the cyanovirocell intracellular response increased, both at the transcriptional and metabolic levels, and was greatest toward the end of the infection cycle. Expression of phage structural genes at that time suggests that phage S-SSM5 was utilizing such host resources for building virions. Functionally, the protist-treated cyanovirocells were more stressed, more P-limited, and enhanced their protein folding and energy acquisition capabilities—observed in the over-expression of central C metabolism genes and increased photosynthetic efficiency. Metabolically, cyanovirocells increased their nucleotides and amino acids, again suggesting protist presence places a higher metabolic demand on infected cyanobacteria. The extracellular metabolome may also reflect such stress as well as prokaryote-eukaryote competition for nutrients given that when the protist was present the resources decreased.

Though only a first step toward mechanism, these findings highlight key areas for future hypothesis-driven experimental work as follows. To analyze the impact of the exometabolites on protist–prey interactions, microfluidic chemotaxis assays (e.g., [114]) combined with live cell video microscopy could quantify the response of diverse bacteria and protists to virocell-released exometabolites and provide an alternative to bulk population-based approaches [21, 22, 107]. In addition, to examine which microbes benefit from the exometabolites and to quantify nutrient utilization, generation, and exchange, stable isotope labeling of virocells, followed by a suite of methodological approaches such as nanoSIMS for spatially resolved compound flux visualization [115], stable isotope probing [116], or quantitative fluxomics [117] could be employed. Finally, the metabolites presented here offer a catalog of specific molecules to target via rapidly advancing mass spectrometry approaches [118], which, along with Bioorthogonal Noncanonical Amino Acid Tagging [119–121], would enable the discovery of virocell-derived signaling molecules, chemoattractants, and chemorepellents—all of which could have profound implications for cell–cell interactions and food web dynamics. As virocell studies mature, a grand challenge for the field will be to incorporate virocells into synthetic microbial communities [122] and ecosystems [123] along with applying advanced mathematical modeling [124]. These added efforts will enable the community to bridge the gap between model organism studies and observational accounts of complex natural communities.

## Conclusions

A decade has passed since the marine scientific community was “dared” to examine interactions between viral infection and protists [17]. Using *Synechococcus* and their viruses, identified as key predictors of biological carbon flux [39], we mechanistically explored interactions among cyanobacteria, cyanovirocells, and protists. We found that cyanovirocells are not the same with and without the protist, such that even low protist abundance increases the energy (e.g., ATP) and resource (e.g., reducing power, phosphate, nucleotides, and amino acids) demand of cyanovirocells relative to protist-free cyanovirocells, especially toward the end of the infection cycle. Here we take a first approach to understanding how the presence of a protist impacts cyanovirocells, not how cyanovirocells respond to protist predation given that the low protist abundance would be a limitation for the latter endeavor. Future approaches with higher protist numbers may investigate how cyanovirocells respond to predation or even how different virocells respond to different predators. While ecosystems biology modeling is in its infancy [124–127], experimental findings like those here are critical to provide baseline data needed to help incorporate viruses and other important top-down predators into our understanding of ecosystem processes.

## Supplementary information

Supplementary information

Supplementary dataset 1

## Data Availability

Raw data, calculations, and statistical tests are available in the Supplementary Dataset, which has been deposited, along with the scripts used in the ‘omics analyses, the metabolomics quality control data, and the genomes used, in the public repositories Cyverse (https://datacommons.cyverse.org/browse/iplant/home/shared/iVirus/Cyano_phage_grazer_omics/) or GitHub (https://github.com/simroux/Metabolomics_CyanophageGrazer). Supplementary files also include expanded methods and analyses for culture conditions, photosynthetic measurements, transcriptomic analyses, LC-MS metabolomics procedures and analyses, choice of protist, protist–prey contact calculations, enumeration of organisms, phage titer determination, and further results and discussion on transcriptomics and metabolomics findings.
