# Supplementary material for: Protist impacts on marine cyanovirocell metabolism
Source: ISME Commun. 2022 Oct 1;2:94. doi: 10.1038/s43705-022-00169-6 (PMC9723779; doi:10.1038/s43705-022-00169-6)
Supplement: Supplementary file 1 — Supplementary information [file 43705_2022_169_MOESM1_ESM.docx]

**Protist impacts on marine cyanovirocell metabolism**

Cristina Howard-Varona, Simon Roux, Benjamin P. Bowen, Leslie P. Silva, Rebecca Lau, Sarah M. Schwenck, Samuel Schwartz, Tanja Woyke, Trent Northen, Matthew B. Sullivan, Sheri A. Floge

CONTENTS:

Supplementary Methods

Supplementary Text

Supplementary Figures

Supplementary Tables

Supplementary Figure Legends

Supplementary References

***Supplementary Methods***

***Enumeration of phages and cyanobacteria***

Phages and cyanobacteria were enumerated using a BD FACSCanto flow cytometer equipped with a 40 mW solid-state laser (488 nm emission). Phage samples were thawed at room temperature, serially diluted in 0.2 μm-filtered Tris-EDTA (TE) buffer pH 8.0 (Invitrogen, cat. no. AM9858), stained for 10 min in the dark with SYBR Green I (Molecular Probes, cat. no. S7563) at 0.5× concentration and discriminated based upon green fluorescence (1). Cyanobacteria samples were thawed at room temperature, serially diluted in autoclaved 0.2 μm-filtered seawater and discriminated based upon chlorophyll *a* and phycoerythrin fluorescence. Individual samples were weighed prior to and after flow cytometry analysis to determine volume analyzed and enable concentration calculations. Blank values were obtained for both Tris-EDTA buffer and filtered seawater and subtracted from raw virus and cell counts, respectively. The absence of heterotrophic bacteria in our experimental system was confirmed throughout the course of the experiment by comparing flow cytometric counts of unstained phycoerythrin and chlorophyll-a based cell counts and SYBR-Green I-stained cell counts. Because heterotrophic bacteria will not be evident in unstained samples, we examined flow cytograms for evidence of additional cell populations or differences in cell abundance (assuming potential overlap of cyanobacterial and heterotrophic bacteria in flow cytograms). No evidence of heterotrophic bacteria was found (data not shown).

***Phage titer and multiplicity of infection determination***

Phage titer (number of infectious phages per mL) was determined using the most probable number (MPN) or end-point dilution method (2). Experimental phage stocks were diluted 10^-4^ to 10^-10^ in sterile SN media in a clear 96-well plate with 12 technical replicates per dilution (virus dilution plate). In three separate additional 96-well plates, 150 μL sterile SN media was added to each well. A total of 30 μL of mid-exponential phase *Synechococcus* strain WH8102 grown at 20^o^C and 60 μmol photons m^-2^ s^-1^ on a 14:10 light:dark cycle was added to all wells except for a one column used for media blank measurements. A total of 20 μL of each virus dilution was added to each well, excluding one row used as a negative control for viral infections (to confirm adequate host cell growth in absence of viruses), for final virus dilutions of 10^-5^ to 10^-11^. A total of 200 μL of liquid was present in each well. Plate edges were sealed with parafilm and placed under experimental growth conditions. *Synechococcus* cell density was monitored using phycoerythrin fluoroscence measured with an Appliskan fluorescence plate reader using 485 nm excitation/ 590 nm emission at 0, 1, 2, 3, 4, 6, 7, 9 days post-infection. Infective phage concentration was estimated from the proportion of lysed wells using the MPN_ver6.xls Excel spreadsheet described in (3), corrected for the final volume of 200 μL (see Supplementary Dataset). The multiplicity of infection (MOI) was calculated as the ratio of infective phage to *Synechococcus* host cells during the initial 1 h infection step.

***Rationale for the choice of protist and its concentration***

We chose the protist *Oxyrrhis marina* because it is an extensively characterized model marine protist (reviewed in (9)). We aimed to use a ratio of protist: *Synechococcus* based on the literature available prior to the time of conducting these experiments, in 2015. In (10), a ratio of dinoflagellates: *Synechococcus* of 1:2×10^7^ was reported, and (11) recorded *Oxyrrhis marina* concentrations of 0.3 - 122 cells per mL, but did not report *Synechococcus* concentrations. Based on these sparse data, we proceeded with 43 *Oxyrrhis marina* cells per mL, which corresponds to a ratio of 1:4.6×10^5^ *O. marina: Synechococcus,* and is thus above that reported in (10). A more recent publication measuring dinoflagellates and *Synechococcus* abundances in the surface ocean (12) noted highly variable dinoflagellate: prey ratios, from less than 1:6.2×10^4^ up to 1:7.2×10^3^, demonstrating that protist-to-prey ratios can be highly variable in nature. Previous studies (22) have reported *Oxyrrhis marina* ingestion rates on *Synechococcus* WH8102 of 1-6 prey cells per protist per hour over short (<2 h) time scales. We observed 61 ± 10% and 50 ± 5% of the total protist population actively feeding in the cyanobacteria with protist and cyanovirocells with protist treatments, respectively (Supplementary Dataset, tabs 3 and 4). Thus, at the protist density used (43 *O. marina* cells mL^-1^), with roughly half the population feeding and a maximum feeding rate of 6 cyanobacteria per hour over ~10 h of the experiment, the maximum amount of prey cells consumed is estimated to be 1.5×10^3^ mL^-1^ (22). *Synechococcus sp*. starting concentration were 2.2±0.1×10^7^ and 1.7±0.3×10^7^ cells mL^-1^ in the cyanobacteria with protist treatment and cyanovirocells with protist treatment, respectively (Ave±SD; See Supplementary Dataset). Thus, with the high prey: protist ratios, protists would not significantly reduce *Synechococcus* population levels at these cell concentrations.

We observed that *Synechococcus* cells formed aggregates (defined as ≥10 cells), with significantly fewer aggregates mL^-1^ in the cyanovirocells with protist treatment (238 ± 55 aggregates mL^-1^) than in the cyanobacteria with protist treatment at 4 h post-infection (645 ± 163 aggregates mL^-1^) (t-test; *p* value<0.05; Supplementary Dataset). Prior work has demonstrated that *O. marina* ingestion rates of 4 μm beads are 4× higher than that on 1 μm beads (24) and the size of cell aggregates observed in our experiment were larger than 4 μm in diameter. We interpret these data to suggest that WH8102 aggregates in our experiment could lead to better capture by *O. marina*. Phages can alter *Synechococcus* cell surface structures or induce chemical cue release (25,26), which could influence aggregate formation. Protists generally exhibit size-selective feeding, thus aggregation could alter protist-prey dynamics. However, since the other treatments were not assessed, this remains an area for future study.

Finally, *O. marina* has the ability to either contact and ingest or contact and reject prey cells (27) based on cell surface properties during the prey processing feeding stage (28). Thus, non-ingested *Synechococcus* cells that have been in contact with *O. marina* could exhibit stress even at low protist-to-*Synechococcus* ratios, as previously shown for phytoplankton prey and zooplankton predators with predator: prey ratios of 0.1:4×10^6^ (29) and 1: 1×10^5^ (30). Infochemicals are integral to protist-photoautotroph interactions (31,32) and exposure of prey cells to protist-derived infochemicals (i.e. cell culture filtrates) elicit phytoplankton defense responses including morphology changes (33,34), chemical composition (35) and fleeing behaviors (36,37).

***Quantification of grazing pressure***

*Oxyrrhis marina* samples were thawed at room temperature, incubated 12 min with wheat germ agluttinin, Alexa Fluor 488 conjugate (ThermoFisher Cat. No. W11261) at a concentration of 5 μg mL^-1^, filtered on to a 5 μm polyester (PETE) membrane filter (Sterlitech Corp. cat. no. PET 5025100), rinsed 3× with 5 mL sterile, 0.02 μm-filtered seawater and allowed to air dry before mounting. Filters were mounted onto slides using Prolong Diamond Antifade Mountant (Invitrogen cat. no. P36961). Samples were excited with a 488 nm Argon laser on a Zeiss LSM 710 confocal microscope. Images were obtained with a 63× (1.4NA) oil objective at Nyquist optimized pixel densities (1584 × 1584) for detailed observations, or a 10× (0.45NA) objective for cell counting. All imaging data were collected using spectral fingerprinting method in lambda mode configuration to optimize differentiation between wheat germ agglutinin (WGA)-stained protist and cyanobacteria phycoerythrin autofluorescence with linear unmixing. Reference spectra for unmixing were created for 488 Alexa tagged WGA and 488 excited phycoerythrin autofluorescence using isolated signal sample cultures with resulting spectra well approximating expected/published emission curves. For cell counts, each filter was imaged at three different locations with a total filter area of 9.72 mm imaged using tiling arrays and z-stack acquisition (9 μm step size) to collect a total of 27-54 μm of sample depth.

***Quantification of protist-prey encounter rates***

Encounter between the *O. marina* protist and the *Synechocococcus* WH8102 cyanobacterial prey depends upon the protist detection radius (r_detect_), the prey particle radius (r_prey_) and the average swimming velocity of both protist (μ_protist_) and prey (μ_prey_). As per (4), the encounter kernel is defined as:

β = π(r_detect_ + r_prey_)^2^(μ^2^_protist_ + μ^2^_prey_)^1/2^

Here, we define the radius of protist detection based on experimental work by (5) in which *O. marina* was reported to have a helical swimming path with a 44 - 80 μm helical width (r_detect_) and a mean forward displacement velocity (μ_protist_) of 400 - 700 μm s^-1^. Because we did not observe any *Synechococcus* motility, to be conservative, we set μ_prey_ = 0 to indicate stationary prey. As per (7), the encounter rate is equal to the encounter kernel multiplied by the concentration of both protist (C_protist_) and prey (C_prey_):

E = βC_protist_C_prey_

Despite ample experimental evidence that *O. marina* exhibits chemosensory capabilities that facilitate prey location and increase encounter frequency (reviewed in (8)), we did not include such parameters in our calculations, thus those reported here are a conservative estimate of encounter rates. The fraction of the *Synechococcus* population 'encountered' by the protist was calculated for each sampling time point by a simple division of total possible encounters/ total number of *Synechococcus* cells and does not include estimates of possible repeat or multiple encounters between the protist and a single *Synechococcus* cell. See Supplementary Dataset for calculations.

***Transcriptomics read mapping, counting, and coverage calculation***

Filtered reads were mapped to cyanophage, cyanobacteria, and protist in a two-step process as follows. First, reads were mapped to the genes predicted from the complete genomes of *Synechococcus* *sp* WH8102 (NC_005070.1) and *Synechococcus* phage S-SSM5 (NC_015289.1), and to genes predicted from whole transcriptome sequencing of *Oxyrrhis marina* CCMP1795 (SRX554017), using BBmap v35.85 (options ambiguous = random) and a threshold of 99% nucleotide identity (i.e. reads mapped with more than 1 mismatch were considered unmapped). Genomes were downloaded from NCBI on 18-August-2015. Second, unmapped reads were then mapped back to the complete genomes of *Synechococcus* *sp* WH8102 and S-SSM5 with using BBmap v35.85 (options ambiguous = random). Read counts were then calculated for each gene using both mapping steps such that the final read count of a gene totaled the number of reads mapped to the gene in the first step (gene-specific mapping) plus those mapped in the second step (genome-wide mapping). Coverage of each organism’s genome was calculated as number of reads mapped multiplied by the read length and divided by the organism’s genome length (see Supplementary Dataset). For *Synechococcus sp* WH8102, read counts mapping to non-rRNA were used in the analyses. Reads were stored in the Ohio Supercomputer Center (13).

***Transcriptomics analyses***

Cyanobacteria and phage read counts were normalized via edgeR (14) in Bioconductor using functions calcNormFactors and estimateDisp, and then transformed into RPKM (15) with rpkm function using log_2_- and prior counts of 0.25. To validate the quality of the biological replicates, phage and host samples were correlated using their log_2_RPKM values and visualized with pheatmap in R (https://cran.r-project.org/web/packages/pheatmap/index.html). Replicates with R^2^ values ≥0.9 were kept for downstream analyses.

For temporal analysis of phage gene expression, log_2_RPKM values were first scaled across protist with phage and protist-only treatments. These values were used for heatmaps and temporal plots (with the pheatmap and ggplot2 (16) packages in R, respectively). Then, the optimal number of clusters was estimated using the benhur and clusterComp functions from the clusterStab package (17). Then, k-means clustering was computed with the obtained optimal number of clusters using the kmeans function from the R stats package. Genes were assigned to individual temporal transcriptional dynamics clusters with the help of the k-means results, hierarchical clustering with heatmap, as well as visual inspection of temporal dynamics generated with the facet_wrap function in R. The temporal clusters were early, when genes peaked at the 3.6 and/or 10-11.9 h post infection; middle, when genes peaked before 6.2 h post infection; and late, when genes peaked at 6.2 h and 8.3 h post infection. To calculate differences of phage gene expression levels between the phage-only and protist with phage treatments, Pearson correlation was calculated between all samples, and visualized with a scatter plot. To calculate differences in phage gene temporal dynamics between treatments, genes were first clustered into temporal expression patterns and when the peak of expression (which marked phage genes belonging to early, middle or late clusters) significantly differed between treatments, as calculated via t-statistic (p<0.05).

For host genes, differential expression (DE) analysis was conducted separately between control and each treatment (cyanobacteria with protist, cyanovirocells only, and cyanovirocells with protist), using glmQLFTest from the edgeR package, with Benjamini-Hochberg correction. The previously edgeR and log_2_RPKM normalized data served as input. Genes with *p* values < 0.01 and false discovery rate < 0.05 were considered DE. Fold change (log_2_FC) was calculated as the expression difference between treatments and cyanobacteria only control. Gene overexpression is considered when log_2_FC >0 and underexpression when log_2_FC < 0. The log_2_FC values were used as the input for the heatmaps, which were generated with pheatmap. Finally, significant host transcripts and metabolites were assigned to metabolic pathways using the Kyoto Encyclopedia of Genes and Genomes (KEGG) ([www.genome.jp/kegg/](http://www.genome.jp/kegg/)) and EcoCyc (18).

***Metabolomics data generation***

Endo-metabolites were extracted from cells collected on 0.2 μm PES filters (#PES0247100, Sterlitech) by adding 1 mL of ice-cold methanol containing 10 µg mL^-1^ 4-(3,3-dimethyl-ureido)benzoic acid (DUBA, #CDS014672, Sigma) as an extraction standard control. Samples were sonicated 15 min in a water bath sonicator and centrifuged 3 min at 3,000 *g* to pellet cell debris. Supernatants were transferred to new tubes, dried in a SpeedVac (SPD111V, Thermo Scientific), and stored at -20ºC. Exo-metabolites were desalted and extracted from filtrate using solid phase extraction cartridges (Bond Elut PPL cartridge, 6 mL, 500 mg, #12255001, Agilent). PPL cartridges were pre-equilibrated with 1 mL methanol (3×) followed by 1 mL water (5×), then air-dried to expel water. Samples with 5 mL filtrate were acidified by adding 100 µL of 6 M HCl, then flowed through the PPL cartridge. Cartridges were rinsed with 1 mL of 0.01 M HCl (twice) then air-dried. Metabolites were eluted by rinsing with 1 mL of methanol containing 10 µg mL^-1^ DUBA (twice) and 1 mL of acetonitrile (twice), after each the eluent was collected into a 5 mL tube. Extracts were dried in a SpeedVac and stored at -20ºC.

Extracts were resuspended in 110 µL methanol-containing internal standards (d4-lysine, 10 µg mL^-1^, #616192; 13C-15N-phenylalanine, 5 µg mL^-1^, #608017; 2­Amino­3­bromo­5­methylbenzoic acid, 1 µg mL^-1^, #R435902; 9­anthracene carboxylic acid, 2 µg mL^-1^, A89405; 3,6­dihydroxy­4­methylpyridazine, 5 µg mL^-1^, #668141; d5-benzoic acid, 10 µg mL^-1^, #217158 – Sigma), then centrifuge-filtered using a 0.22 µm hydrophilic PVDF (Multiscreen GV filter plate, #MSGVS2210, Millipore) into a 96-well autosampler plate. LC-MS was performed using an Agilent 1290 UHPLC coupled to a Thermo QExactive (Thermo Scientific, San Jose, CA) mass spectrometer (MS). Full MS spectra was collected in centroid format from *m/z* 70-1050 at 70,000 resolution, with MS/MS fragmentation data acquired using stepped 10, 20 and 30 eV collision energies at 17,500 resolution. Chromatography was performed on 2 µL injections into a HILIC column (SeQuant ZIC-pHILIC, 5µm polymer, 150×2.1 mm, #150460) warmed to 40ºC at a flowrate of 0.25 mL min^-1^ equilibrated with 100% buffer B (95 % v/v ACN, 5mM ammonium acetate in water) for 1.5 min, diluting buffer B down to 50% with buffer A (5mM ammonium acetate in water) over 23.5 min, down to 40% B over 3.2 min, down to 0% B over 6.8 min, and isocratic elution in 100% buffer A for 3 min. MS source settings included a sheath gas flow rate of 55 (au), auxiliary gas flow of 20 (au), sweep gas flow of 2 (au), spray voltage of 3kV and capillary temperature of 400ºC. An injection blank of 100% methanol was run between each sample injection. Raw data is available for download at <https://genome.jgi.doe.gov/portal/>.

Metabolites were identified by comparing retention time (RT), exact mass and fragmentation patterns from experimental sample spectra with that of metabolite standards run using the same LC-MS methods. LC-MS data was analyzed using custom Python code (19). Each detected feature (unique *m/z* coupled with RT) was assigned a score (0 to 3) representing the level of confidence in the metabolite identification. Metabolites given a positive identification had detected *m/z* </= 5 ppm or 0.001 Da from theoretical as well as RT </= 0.5 min compared to a pure standard run using the same LC-MS method. A metabolite with the highest level of positive identification (score of 3) also had matching MSMS fragmentation spectra compared to either an outside database (METLIN) or internal database of standards ran using the same methods. MS/MS mismatches to the standard invalidated an identification. The final list of identified metabolites was refined by comparing peak height, *m/z* and RT between all pairs of compounds detected across the positive and negative modes for each experiment (endo- and exo-metabolites). When metabolites were identified in both positive and negative ion modes for a given sample, the highest value of peak height or peak area was used for analysis. Duplicates were identified as pairs of compounds with high correlation in peak height (Pearson correlation ≥0.95), an absolute difference in *m/z* < 0.005 (when in the same polarity mode), and absolute difference in RT < 0.1. For these, only one of the pair was retained for final analysis, with preference given to the one with highest confidence of identification.

***Metabolomics data analysis***

For determining which metabolites were enriched or depleted in the treatments relative to the cyanobacteria only control, the threshold of FC≥0.1 and FDR≤0.05 was applied, as explained in Materials and Methods. This threshold was changed for cases where an internal standard’s abundance (which were added equally to the samples) was significantly different between treatment and control (specifically “Media T2 cyanobacteria only vs cyanovirocells”, “Media T3 cyanobacteria only vs cyanovirocells with protist”, “Media T6 cyanobacteria only vs cyanovirocells with protist”). For these cases, the FC of the internal standard was added to the original 0.1 threshold, leading to thresholds of 0.44 in Media T2 cyanobacteria only vs cyanovirocells, -0.26 for Media T3 cyanobacteria only vs cyanovirocells with protist, and -0.5 for Media T6 cyanobacteria only vs cyanovirocells with protist. These new thresholds were only applied to compounds whose FC had the same sign of the internal standard, e.g. for Media T2 cyanobacteria only vs cyanovirocells, a compound with a FC of 0.3 and FDR < 0.05 would not be considered significant (because lower than the new threshold), however compound with a FC of -0.3 and FDR < 0.05 would be considered significant. Heatmaps and plots were generated using pheatmap and ggplot2, with compounds clustered automatically based on FC, and samples sorted by time points.

***Transcriptomics and metabolomics comparative analyses***

1. *Saturation curve*

To verify that coverage depth was sufficient to represent the entire *Synechococcus* and phage transcript profiles, saturation curves were generated by subsampling the reads of each individual library. Briefly, a custom Perl script was used to select random subsamples of reads mapped to each genome, first 10 to 100 reads by increment of 5, then 100 to 10,000 reads by increment of 100, and finally 10,000 to 1,000,000 reads by increment of 50,000. For each subset, the number of genes “seen” (i.e. with at least 1 read mapped) was tallied, and used to generate the rarefaction curve of the corresponding sample with ggplot2 (16) and a palette from ggpubfigs (20).

1. *Re-analysis of metabolomics data with different cutoffs*

In order to evaluate how cutoff decisions may influence the number of compounds detected as significantly changing between treatment and control samples, the metabolomics data were re-analyzed following cutoffs and metrics used in (21). The same metrics for metabolite abundance (peak height) and the same statistical test (t-test with Benjamini-Hochberg adjusted *p* value) was used as in our baseline analyses, however the values were log_2_-transformed instead of log_10_-transformed, and compounds with an absolute abundance fold change ≥ 0.5 and false-discovery rate ≤ 0.1 were considered as significantly different between treatment and control. This procedure was applied to each time point separately.

***Supplementary Text***

1. ***The specifics of phage transcriptional dynamics and the phage genes that have altered transcription in the presence of protist.***

The 5 genes that shifted temporal regulation in the presence of protist included a tRNA-Valine gene (SSM5_020), a ferrochelatase (SSM5_043), 2 genes of unknown function (SSM5_080 and SSM5_082), and the terminase DNA packaging small subunit enzyme (SSM5_105) (**Fig. 2b, Supplementary Fig. S3**). Presence of tRNAs is common in phage genomes and speculated to complement the host translational machinery by encoding for tRNAs of the most used phage codons to, in turn, increase fitness (38). In cyanophages specifically, *Synechococcus*-infecting phages, as well as T4-like Myovirus phages, are suspected to especially benefit from encoding for their own tRNA genes due to the greater codon usage bias associated with the larger GC content of *Synechoccous* over *Prochlorococcus*, and of the broader host range T4-like phages than the T7-like podophages (39). Thus, modifying expression of tRNA genes may be a strategy to enhance S-SSM5 translation during protist predation. Additionally, ferrochelatase is an essential enzyme for synthesizing heme, a tetrapyrrol involved in nearly all metabolic pathways, including photosynthesis (40). A shift in ferrochelatase expression may reflect the need to fine-tune host metabolic functions, including photosynthesis, in the presence of a protist. Finally, of the two terminase subunits in T4-like phages, the small one is required to position the large one to the prohead and translocate DNA for a “headfull” package (41,42). Change in terminase expression in the presence of a protist suggests phages actively regulate the DNA packaging machinery in response to additional stressors, such as the presence of potential predators. Altogether, these results provide a window into the cyanophage genes that are most critically regulated at the expression level in response to protists.

1. ***Comparison of the host transcriptional response between our study and Doron et al 2016’s.***

Given that *Synechococcus* WH8102 displayed a smaller transcriptional response to S-SSM5 than to Syn9 reported by (43), we analyzed the differences between our study and theirs, focusing on RNA-seq sequencing depth and genome coverage as well as experimental design, keeping in mind that Doron et al (43) performed the host transcriptome analysis from microarray data due to their low RNAseq signal for the host.

First, on sequencing depth and genome coverage. The sequencing depth for WH8102 non-rRNA regions from (43) ranged between 1.31×10^5^ and 2.84×10^6^ (black bars, **Supplementary Fig. S4a**). In our study the sequencing depth ranges are 2.3×10^5^ - 7.88×10^6^ (for S-SSM5 infection alone; red bars, **Supplementary Fig. S4a**) and 7.78×10^5^ - 4.76×10^6^ (S-SSM5 infection with the *O. marina* protist; blue bars, **Supplementary Fig. S4a**). With this sequencing depth, the average host genome coverage in the (43) study is either 20.5× or 41× depending on whether the Illumina sequencing data in Table S3 derives from 50 bp or 100 bp calculations, respectively (we were unable to find this information in their methods, supplementary information or website) (**Supplementary Fig. S4b**). This average coverage is calculated considering all time points, and it is low due to sequencing depth decreasing over time (**Supplementary Fig. S4a)**. In contrast, in our study the sequencing depth does not decrease over time (**Supplementary Fig. S4a**) and the average host genome coverage across all our sampled time points is 295× and 155× in the cyanovirocells and the cyanovirocells plus protist treatments, respectively (**Supplementary Fig. S4b**, Supplementary Datset tab 20). Finally, a saturation curve made following a recent publication on RNA-seq “best practices” (44) revealed that our sequencing data is sufficient to detect all host genes (**Supplementary Fig. S4c**). Together, these analyses show that the RNA-seq data collected here was adequate for analyzing host transcriptional responses in all treatments.

Second, on the differences in the number of differentially expressed (DE) host genes in response to phage infection. The (43) study reports "a marked decline of the vast majority of host genes (>90%)" 0.5 to 1 hour post infection and focuses on the 130 remaining genes that in WH8102 respond to Syn9 infection from the beginning to the end of infection, which are termed “host-response” genes. In our system we detected 48 and 216 host-response genes from 2 – 12 h post infection for the cyanovirocells only and the cyanovirocells with protist treatments, respectively. Because (43) also reports that the housekeeping gene *rnpB* had unchanged expression in the WH8102 infected and uninfected samples, we analyzed the pre-normalization log2RPKM values of *rnpB* in our dataset to ensure that there similarly were no expression changes across treatments, thus providing more solid ground for calling out the host-response genes after RNA-seq normalization. Indeed, *rnpB* was not significantly different in any treatment (protist-treated cells, cyanovirocells only, or cyanovirocells with protist) relative to the untreated control (**Supplementary Fig. S4d)**, whereas both the over-expressed (**Supplementary Fig. S4e)** and the under-expressed (**Supplementary Fig. S4f)** host-response genes had significantly different log2RPKM values in both virus treatments, the cyanovirocells only and the cyanovirocells with protist (t-test, *p* value<0.05). This analysis reassures that, unlike housekeeping genes which have unchanged expression across treatments, the genes we report as significantly changing can indeed be termed host-response, mirroring the (43) study.

Two possible explanations for the differences in DE host genes observed in (43) vs our study are the different phages used and the experimental setup for transcriptomics analyses, as follows. While the phages from (43) (Syn9) and our study (S-SSM5) are both T4-like Myoviruses, they are different enough that the host transcriptional response would be different. A growing body of literature suggests that there is phage-specific metabolic reprogramming for cyanobacteria (43,45), marine heterotrophic bacteria (46,47), and pathogenic heterotrophic bacteria (21,48). This means that the same host will have different responses against different phages.

Further, the experimental design is different in terms of multiplicity of infection, phage addition, and when RNA was sampled. The last point is an important difference between the studies, because the different sampled time points would lead to capturing different host responses between (43) and that reported here. Specifically, (43) analyzed the host transcriptional response between 0 h and 2 h post phage addition, whereas we sampled between 2 h and 12 h, thus missing the early response that (43) captures. This is an important difference because the host is expected to have a large transcriptional response early when infected with T4-like phages, given that the canonical coliphage T4 massively reprograms the host towards phage reproduction immediately after infection and requires the host’s machinery to help with that (49,50). Consequently, a large number, perhaps even the majority, of host genes are differentially expressed very soon after infection with T4-like phages, and this has been noted for several T4-like cyanophages with temporally-resolved genome-wide transcriptomics data such as Syn9 (43), P-SSM2 (infecting Prochlorococcus, (51)) and P-HM4 (infecting Prochlorococcus, (52)). Given these findings, it would be expected that, in our study, the largest host transcriptional response to S-SSM5 would also occur before our first sampled time point (2 h). In summary, our study captured a different type of host response than that reported by (43) due to having a different phage and having sampled at later time points, thus complementing (43) findings and providing novel findings regarding cyanovirocell responses to the presence of a protist.

1. ***Comparison of the metabolomic data across similar studies***

The number of metabolites reported here as significantly changing in a treatment relative to the control is lower than that reported by the other temporally-resolved virocell studies (21,53,54). Here we provide a comparison between ours and these other studies (**Supplementary Table S2**). One of the biggest differences is that our metabolomics approach was targeted, meaning that only compounds that were confidently identified by matches to our internal database were considered. Work by (53) was also targeted, but publications by (54) and (21) used untargeted approaches, which aim to capture any chemical feature generated and consequently will report more compounds. When comparing our study against those, the number of total masses that we obtained (n=264) is higher than those in (53) (n=83) and comparable to those reported by (21) (n=375) and (54) (n=377). Further, our significantly changing metabolites represent 4-9% of the 264 masses detected, which are also within the range (0.27-36.8%) reported by (21) in their metabolomic analysis of 6 different phages.

We also differ from the cited studies in the approach to call a metabolite as significantly changing across treatments. We considered peak height and log_10_-transformed it, whereas (54) and (21) (two publications from the same group) log_2_-transformed the peak height, and (53) used peak area, which they manually curated and normalized to cell density, which was impacted during infection (53). Most notably, our approach was more stringent due to the use of 10 internal standards, as we required that a metabolite have a significantly greater change than that of the standard. To test whether our approach was more stringent, we applied the analytical procedures found in (21) to our raw data (since (54) uses the same procedures, and (53) had a manual step and normalized to cell density, which we caution doing because cell lysis decreases cell density and skews the calculations). We obtained 3 to 17-times more significantly changing metabolites (**Supplementary Table S3**, Supplementary Dataset tab 21), confirming our hypothesis that one of the reasons for our lower number of significantly changing metabolites is our stringent analytical approaches. In summary, *i*) our metabolomics approach was targeted, which limits metabolite identifications, *ii*) our total number of masses identified by the mass spectrometer is comparable to recent publications, *iii*) our significantly changing metabolites are within the range reported in another metabolomics study, but are few in number due to our stricter approaches for defining significantly changing metabolites, including the use of internal standards.

1. ***The specifics of cyanovirocell reprogramming of central C metabolism, nucleotide synthesis, photosynthesis and P stress in the presence of a protist***

Evidence of protist impacts on cyanovirocell metabolic reprogramming was evident in the differential expression of host genes (phage auxiliary metabolic genes (AMGs) involved in those metabolisms were similarly expressed with and without protist). Such host genes were greater in number during phage infection with protist (n=23) than without it (n=4). The following explains in more detail what those genes were.

During phage infection, 4 host genes were over-expressed (**Supplementary Fig. S5b**): 2 involved in phosphate (P) stress response (*pstS* and *phoH*), 1 involved in photosynthesis (*hli*), and 2 genes involved in alternative central carbon (C) metabolism pathways – mannose synthesis (*cpsB*) and galactose transformation to glucose (*nagA*) (**Fig. 4, Supplementary Fig. S5, Supplementary Table S3**). All but *nagA* were over-expressed, suggesting that phage-infected *Synechococcus* was metabolically enhanced, not repressed, to incorporate phosphate, protect the photosynthetic machinery, and alter energy acquisition through selectively rerouting C metabolism.

The 4 host genes that were over-expressed with phage were also over-expressed when protist was added (**Supplementary** **Fig. S5**). Gene *nagA* was not DE in the presence of both phage and protist. In total, 23 host genes were over-expressed with both phage and protist, 19 additional to phage infection alone. These included 11 additional photosynthetic genes (*hli,* *cpeT, pcxA, cpeC, ndhO, cydB, petF, ycf3*), 2 additional P-stress genes (*phoA*, *phoB*), 5 additional central C metabolism genes (*cpsB*, *fbaA*, *talB*, *rbcL* and *rbcS*), and 2 de novo nucleotide synthesis genes (*prs* and *ndk*) (**Supplementary** **Fig. S5**). The photosynthesis genes include reaction centers, protection and electron transfer additional to the already expressed phage AMGs (**Fig. 4a**), thus showcasing greater demand for photosynthesis in cyanovirocells co-cultured with protists. Additionally, the alkaline phosphatase *phoA* and the response regulator *phoB* are markers of phosphate stress, together with the transport genes (including *pstS*) expressed by both phage and host (55). Their over-expression only in the presence of phage suggests that the cyanovirocells had a higher P demand when protist was present than when it was absent (**Fig. 4a**). As P acquisition in cyanovirocells has been attributed to the need to replicate phage DNA, these data suggest that such need is greater with protist than with phage alone. Similarly, cyanophage DNA replication, like that of most phages, requires more nucleotides than that provided from recycling host DNA (56), and *de novo* nucleotide synthesis is energetically-costly. The energy for such process is thought to derive from degrading sugars via glycolysis, the pentose phosphate pathway (PPP) and Calvin cycle (57). Various phage AMGs (*zwf, gnd, talC* and *cp12*) and host genes were over-expressed from these expected pathways in cyanovirocells co-cultured with protists. The host genes included *cpsB* (mannose synthesis as input for glycolysis), *fbA* (involved in glycolysis and the calvin cycle), *talB* (involved in the interface between the pentose PPP and the calvin cycle), and *rbcL* and *rbcS*, which are subunits of Rubisco, the signature enzyme of the Calvin cycle (**Fig. 4b**). Cyanophages are known to redirect sugar metabolism through these pathways in order to create reducing power and metabolites such as ribose-5-phosphate (R5P) for nucleotide synthesis (58). The bridging gene between PPP and nucleotide synthesis is *prs*, a host gene that was over-expressed only in cyanovirocells co-cultured with protists, and which transforms R5P to PRPP (5-phospho-D-ribose alpha-1-pyrophosphate), an intermediate in both purine and pyrimidine nucleotide *de novo* synthesis (**Fig. 4c**). In those pathways, the expressed *ndk* gene is promiscuously involved in the terminal steps of adenine, guanine, cytosine and thymine nucleotide synthesis (**Fig. 4c**). Finally, while the virocells alone had no metabolites changing from this pathway, when protist was added there was a significant increase in the purine nucleotide guanine and one of its derivatives, and around the same time of host gene over-expression (**Fig. 3b**).

1. ***Photosynthetic efficiency measurements***

Cyanobacteria obtain energy via light absorption by the light harvesting antennae of photosystem II (PSII). Upon absorption, light energy may take three pathways including photochemistry (i.e., primary charge separation in reaction center II (RCII)), fluorescence, and heat dissipation. Active fluorometry, such as fast repetition rate fluorometry (FRRf), utilizes the complementary nature of the three possible pathways of light energy to quantify the photophysiological state of the cells. Triggering and detecting changes in fluorescence in the dark-regulated state allows derivation of the commonly used parameter F_v_/F_m_, or the maximum quantum yield of PSII, a measure of the maximum fraction of absorbed light energy that can be used for primary charge separation in RCII.

Our data shows that phage infection results in a 35% decrease in overall photosynthetic efficiency as early as 2 h post-infection and further declines up to 64% by 12 h post-infection (**Fig. 4d; Supplementary Fig. S8;** Supplementary Dataset). The current paradigm holds that phages maintain high levels of photosynthetic efficiency in cyanovirocells via expression of viral photosynthesis genes and translation of proteins that actively modulate host photosynthetic electron transport chains, driving enhanced energy flow toward nucleotide biosynthesis (58–60). Despite the apparent differences in F_v_/F_m_, our data also support this paradigm and provide direct evidence for phage-driven enhanced cyclic electron flow and respiration, or deviation of light energy toward ATP production. During cyclic electron flow, plastoquinones are utilized as electron carriers in the oxygen-mediated oxidation of NADPH. Prior studies have utilized multiple turnover (MT) active fluorescence measurements that do not directly detect changes in plastoquinone pool (PQ) quenching, thus F_v_/F_m_ values would remain high even in the presence of PQ reduction (61–68). Conversely, the single turnover (ST) active fluorometry technique, such as that used in this study, has a minimal effect on the redox state of the PQ pool resulting in higher minimal fluorescence (F_o_) and maximal fluorescence (F_m_) and reduced F_v_/F_m_ values in the presence of cyclic electron transfer. While a direct comparison of cyanovirocells with both MT and ST methods is warranted, our fluorescence data (increased F_o_, F_m_ (Supplementary Dataset), and decreased F_v_/F_m_ (**Fig. 4d, Supplementary Fig. S8,** Supplementary Dataset)) suggest phage-induced cyclic electron flow in agreement with prior work. As noted above, multiple phage genes that encode proteins directly impacting light absorption were expressed during infection, including high light inducible protein (*hli*), PSII proteins (*psbA*, *psbD*, *speD*), and plastocyanin proteins (*petE*). Most notably, phage S-SSM5 expressed the gene *ptox* (**Fig. 4**), which encodes plastoquinol terminal oxidase and has been implicated as a mediator of alternative electron flows from PSII (60,69).

Alternatively, cyanobacteria may undergo state transitions wherein light energy is preferentially absorbed by PSII (State 1) or PSI (State 2). Dark-adapted cells in State 2 will exhibit lower F_o_ but F_m_ will remain unchanged, leading to lower F_v_/F_m_ or photosynthetic efficiency (70). Our data show that phage-infected *Synechococcus* populations exhibit both increased F_o_ and F_m_ relative to uninfected controls strongly suggesting that PQ pool reduction rather than state transitions were the cause of reduced photosynthetic efficiency values observed in phage treatments relative to controls.

The photosynthetic efficiency (F_v_/F_m_) of cyanovirocells in the presence of the protist increased by roughly 15% (±5%) just before cell lysis (~5 h post-infection) whereas no increases in photosynthetic efficiency were noted over time in the absence of the protist (3% ± 8%) (**Fig. 4d; Supplementary Fig. S8;** Supplementary Dataset). We propose that this difference lies in the cyanovirocell response to the stress of the presence of the protist and is likely linked to differential expression of host genes (*cpeC*, *cpeT*, *hli* (n=4), *pcxA*, *ndhO*, *cydB*, *petF*, *ycf3*) involved in energy acquisition pathways.

**SUPPLEMENTARY FIGURES**

**

**

**Supplementary Fig. S1: Whole-genome comparison of Syn9 and S-SSM5**. Blastn-based alignment. S-SSM5’s genes are color-coded based on the functions described in alphabetical order.


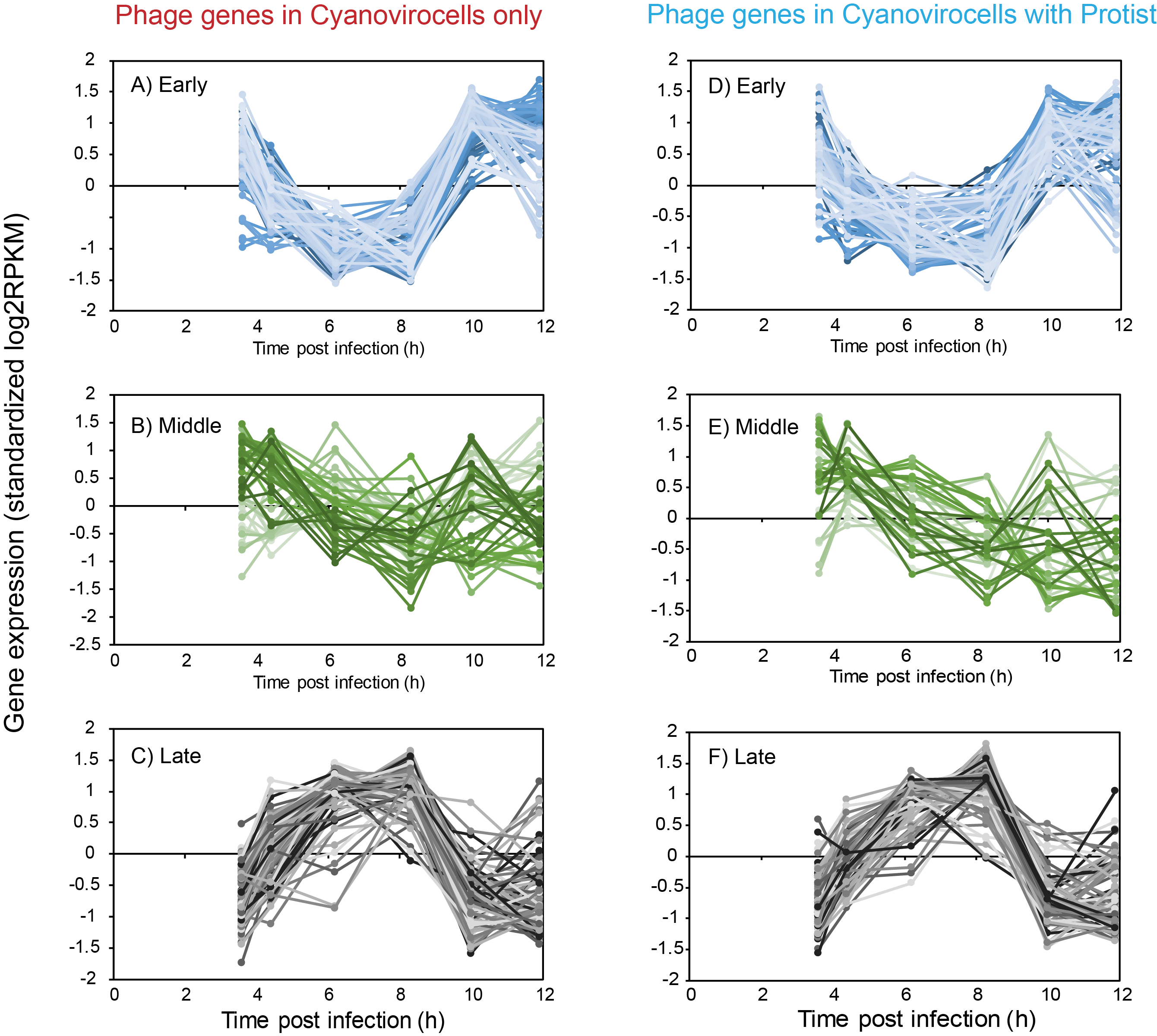


**Supplementary Fig. S2: Phage temporal transcription during infection of *Synechococcus* WH8102 in the presence or absence of the protist**. Represented is the standardized log_2_RPKM values of each gene for each of the temporal clusters, early, middle or late.


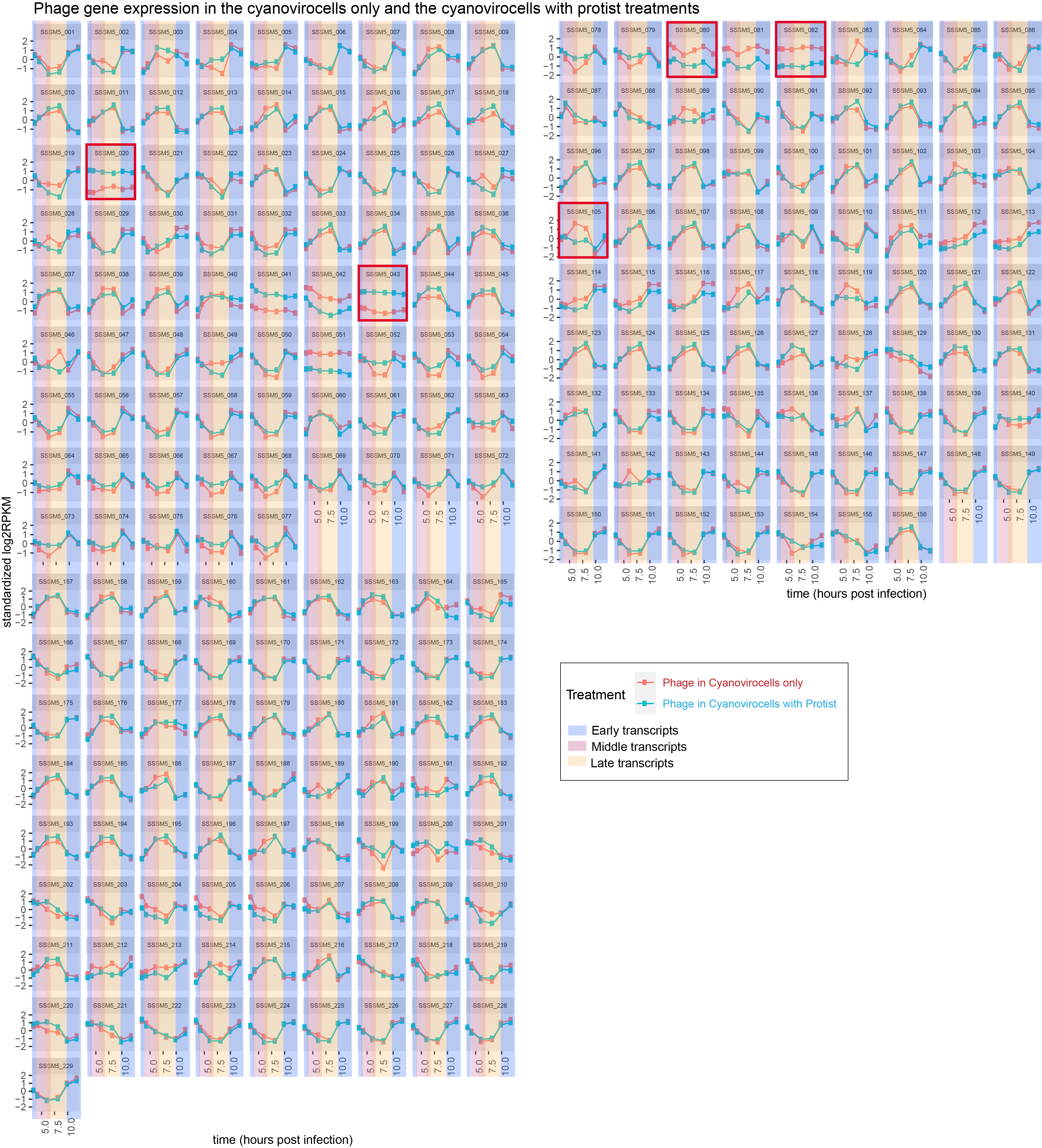


**Supplementary Fig. S3: Temporal expression dynamics of all phage genes in the presence and absence of the protist.** Represented in each graph is each individual phage gene’s expression, measured as standardized log_2_RPKM over time in both cyanovirocell and cyanovirocell with protist treatments. The three colored shades represent when early (blue), middle (red) and late (yellow) transcripts have their peak of expression. Genes that significantly change temporal dynamics between treatments (t-test, p<0.05) are boxed in red.

**
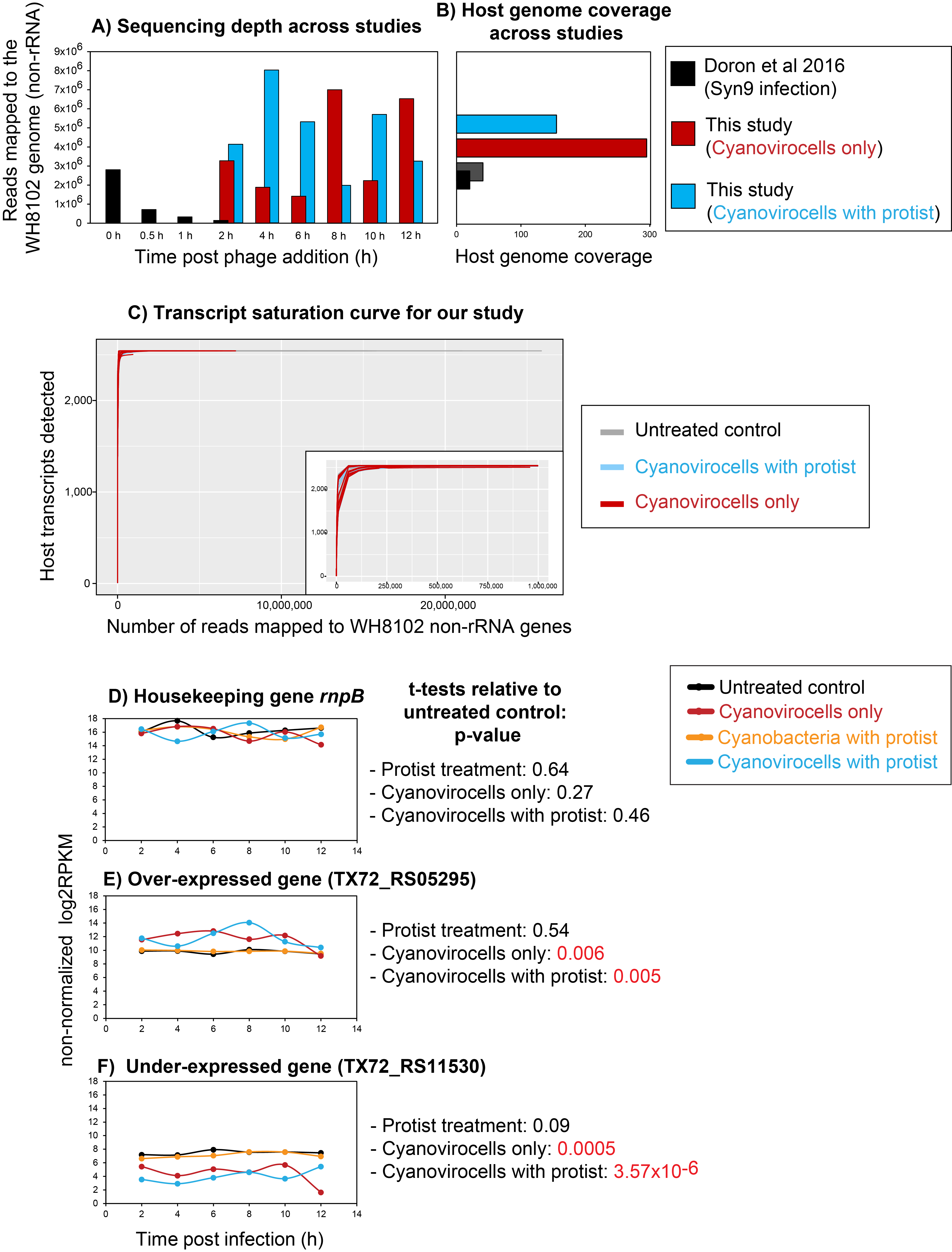
Supplementary Figure S4**: **RNA-Seq data comparison between our study and Doron et al 2016**. a) Comparison of average RNA-seq sequencing depth across our study and (43) at different time points post phage infection (0 h, 0.5 h, 1 h and 2 h for (43) vs 2 h to 12 h in our study). Data for (43) sequencing depth derives from Table S3 of their publication. b) Coverage obtained from RNA-seq data for the host genome in (43) and our study. The two calculated host coverage values for (43) are 20.5× (black) and 41× (dark gray), as explained in the text. c) Saturation curve showing how many host transcripts are detected with the number of reads mapped to the non-rRNA regions of the host genome. Our sequencing depth is sufficient to saturate the transcriptome. Inset: the same type of graph, but a closer look for 1 million reads. D-F) Pre-normalization analysis of three bacterial genes. Plotted is the non-normalized log2RPKM of (D) the housekeeping gene *rnpB*, (E) an over-expressed gene, and (F) an under-expressed gene, over the experimental time course, for all four treatments (uninfected control, protist-treated cells, cyanovirocells, and cyanovirocells with protist). To the right of each plot are the results of the t-tests evaluating if there are significant differences in the log2RPKM of that particular gene for a given treatment (i.e. protist treatment, cyanovirocells only, or cyanovirocells with protist) relative to the control, with *p* values<0.05 marked in red. Housekeeping gene *rnpB* is the only one that shows no significant differences in any treatment relative to the control.


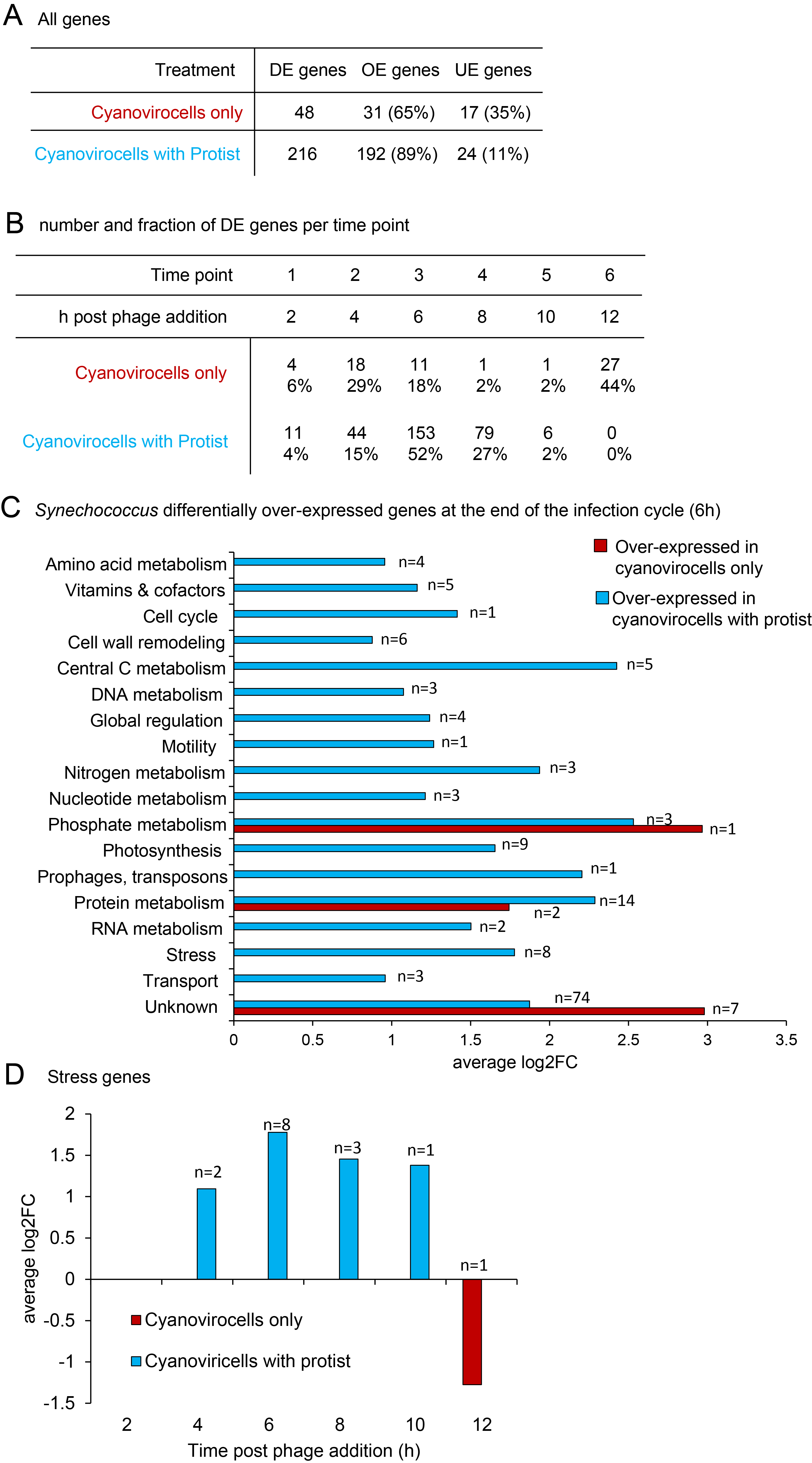


**Supplementary Fig. S5: Cyanovirocell host genes differentially expressed in response to the protist.** A) Number of differentially expressed (DE) host genes, and fraction of over- or under-expressed, in response to phage infection or combined phage and protist predation. B) Number and fraction of DE genes per time point of infection in the virocells with (blue) and without (red) the protist. C). Functional assignment of the host genes that are differentially over-expressed in the virocells relative to the control, at 6 hours after phage addition, in the presence or absence of the protist. Functional categories are determined based on gene function assigned from the NCBI genome. D) Host genes involved in Stress. For C) and D), the plotted values represent the average of the log_2_FC values of the individual genes in each category, with the number of genes next to each bar in the chart.


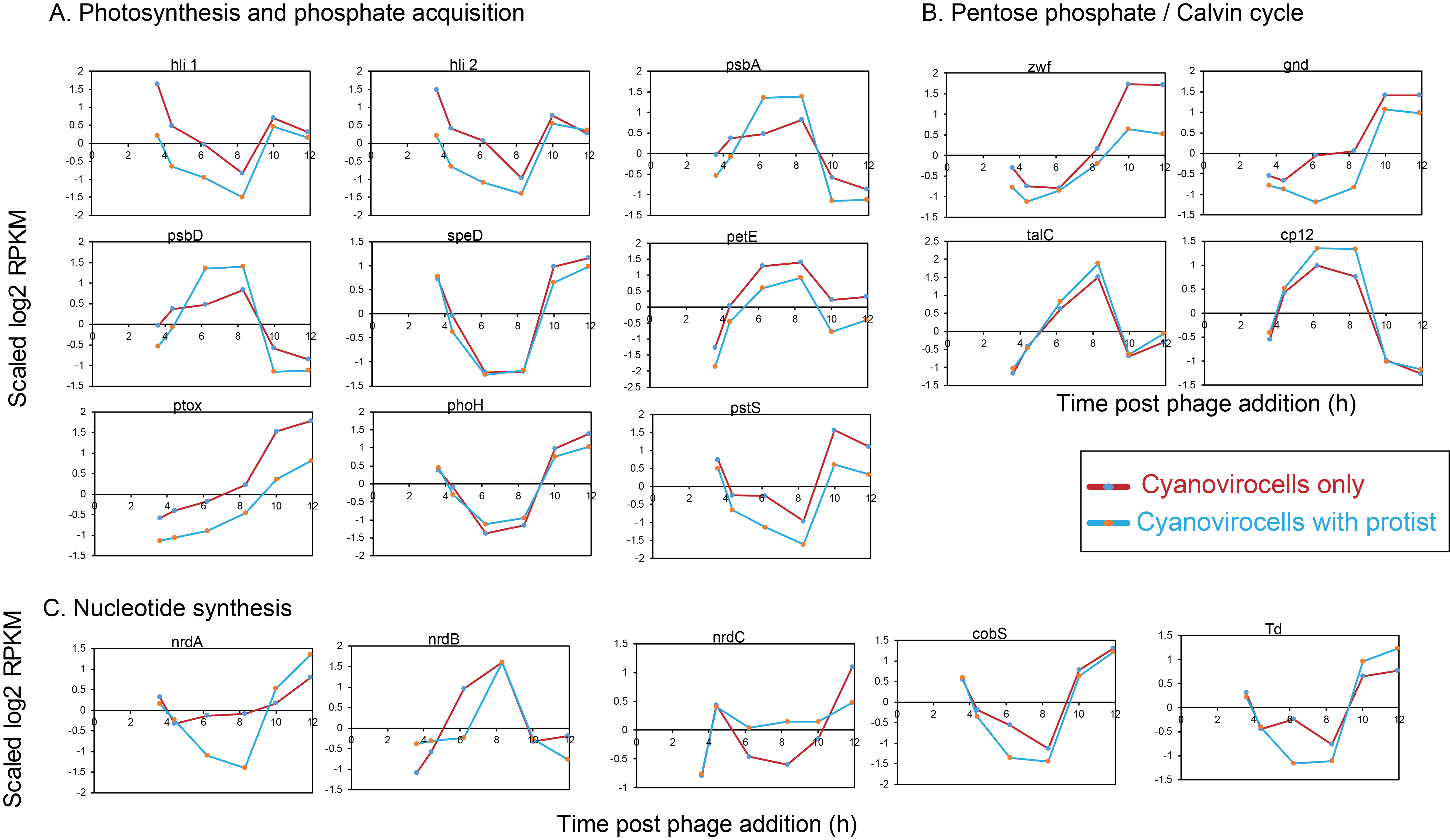


**Supplementary Fig. S6**: **Expression of select phage auxiliary metabolic genes (AMGs) with or without the protist.** Represented is the standardized log_2_RPKM values of each gene over the course of infection with (orange) or without (blue) the protist.

**
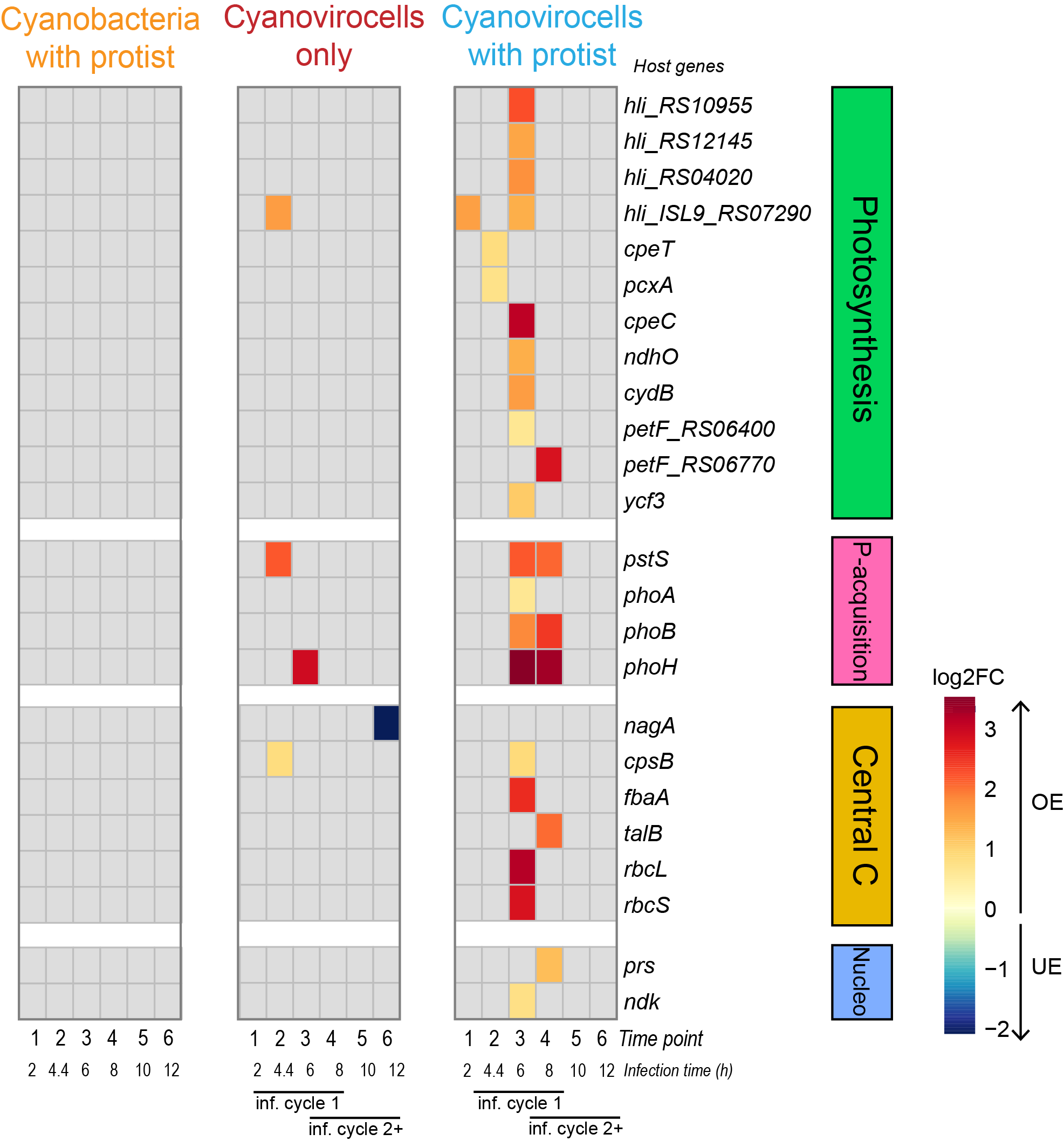
**

**Supplementary Fig. S7**: **Differential expression of host genes involved in the pathways reprogrammed by phage infection.** Pathways shown in Figure 4 and here include photosynthesis, phosphate (P) stress, central carbon (C) metabolism, and *de novo* nucleotide synthesis (Nucleo). OE: over-expressed; UE: under-expressed Abbreviations for the genes and proteins: *hli*: high light inducible; *pcxA*: proton exclusion protein; *cpeC*: phycobilisome; *cpeT*: antenna protein; *ptox*: plastid terminal oxidase; *ndhO*: NADPH quinone oxidoreductase subunit O; *cydB*: cytochrome D ubiquinol oxidase subunit II; *petF*: ferredoxin; *ycf3*: photosystem I assembly protein; *pstS:* phosphate ABC transporter periplasmic binding protein; *phoA*: alkaline phosphatase; *phoH*: ATP-binding protein; *phoB*: DNA-binding transcriptional regulator; *cpsB*: mannose 1 phosphate guanylyltransferase mannose 6 phosphate isomerase; *fbaA*: fructose-1,6-bisphosphate aldolase; *nagA*: N-acetylglucosamine-6-phosphate deacetylase; *talB:* transaldolase; *rbcLS*: rubisco; *prs*: ribose phosphate pyrophosphokinase; *ndk*: nucleoside diphosphate kinase. The time course captures more than one infection cycle: the first one with the phages added at the start of the experiment (“inf. cycle 1”) and subsequent cycles initiated by phages released (“inf. cycle 2+”).


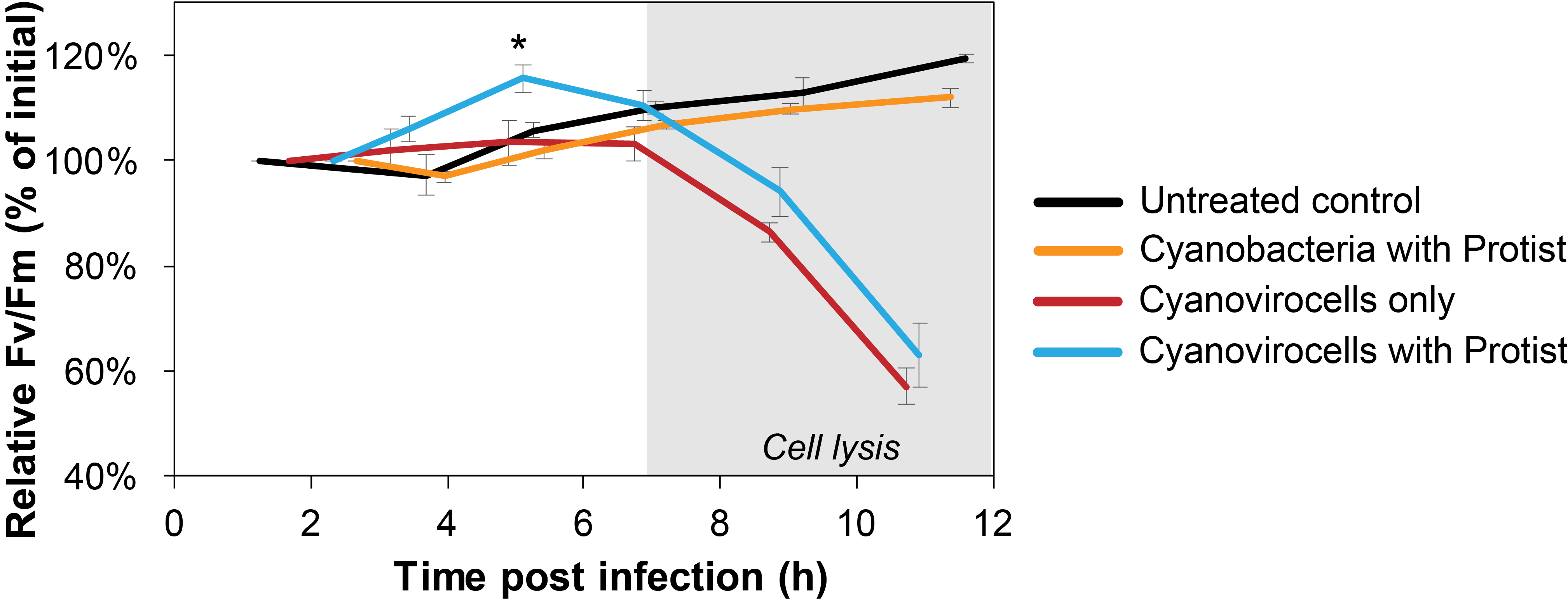


**Supplementary Fig. S8: Photosynthesis efficiency of *Synechococcus* under different conditions, relative to the first time point.** This figure is an extension of Fig. 4D to better show the relative temporal dynamics of the photosynthesis efficiency of each treatment over time. The F_v_/F_m_ values have been normalized to the first time point. While the phage treatment does not significantly increase the photosynthesis efficiency of the cells over time, the phage plus protist treatment does, as indicated by an asterisk (t-test, *p* value<0.05).

***Supplementary Tables***

**Table S1: *Synechococcus* differentially expressed genes and significantly changing metabolites in the presence of the protist, the phage, or both, compared to the untreated control.**

|  | **Cells plus protist** | **Cells plus phage (Cyanovirocells)** | **Cells plus protist plus phage (Cyanovirocells with protist)** |
| --- | --- | --- | --- |
| **Total number of differentially expressed genes** | 0 | 48 | 216 |
| **Over-expressed gene count** | - | 31 | 192 |
| **Under-expressed gene count** | - | 17 | 24 |
| **Significantly changing endo-metabolites** | 0 | 0 | 10 |
| **Enriched endo-metabolite count** | - | - | 8 |
| **Depleted endo-metabolite count** | - | - | 2 |
| **Significantly changing exo-metabolites** | 2 | 23 | 15 |
| **Enriched exo-metabolite count** | 0 | 19 | 11 |
| **Depleted exo-metabolite count** | 2 | 4 | 4 |

**Supplementary Table S2: Comparison of the temporally-resolved, genome-wide metabolomics of phage-host interactions cited in our manuscript against our own study.**

|  | **Ankrah et al 2013** | **De Smet et al 2016** | **Chevallereau et al 2016** | **This study** |
| --- | --- | --- | --- | --- |
| **System** | Marine heterotroph (Sulfitobacter) | Pathogenic heterotroph (Pseudomonas) | Pathogenic heterotroph (Pseudomonas) | Marine cyanobacteria (Synechococcus) |
| **Number of phages** | 1 phage + 1 induced prophage | 6 | 1 | 1 |
| **MS Method** | Targeted | Untargeted | Untargeted | Targeted |
| **Standards** | none | none | none | 10 |
| **Biological replicates** | 2 | 4 | 4 | 4 |
| **MOI** | 4 | 35-100 | 25 | 1.1 |
| **How to obtain Fold-Change** | manually curated peak area, then metabolite area counts normalized to cell density. Then ratio infected to uninfected, log2-transformed. *p* value<=0.05* | log2-fold change of the raw peak height. “cutoffs of abs(log2(fold change))≥0.5 and adjusted *p* value<0.1” | Same as De Smet 2016 | log10-transform peak height, then calculate FC. *p* value <=0.05. Considers FC of significantly changing standards. |
| **Comparing to control** | compared to each respective time point of uninfected? (unclear from methods) | all compared to uninfected time 0 min | Same as De Smet 2016 | compared to each respective time point in untreated (e.g., “T3” in treatment to “T3” in control) |
| **Masses identified** | 83?** | 375 | 377 | 264 |
| **% of masses significantly changing** | 16-73%** | 0.27-36.8% | 18-35% | 4-9% |
|  |  |  |  |  |

*Manual curation of peaks makes it difficult to replicate their procedures. Additionally, normalizing to cell density likely inflates the number of significantly changing metabolites obtained over time, because over their 480 min time course, cells are lysing with their sizable burst (end of phage infection cycle between 60-240 min, Fig. 1A)

**83 reported compounds (Table S3 and S4), 79 of which change significantly (Table S3 and S4). Percentages are based on 83.

**Supplementary Table S3: Number of significantly changing metabolites in our study using De Smet et al 2016’s approaches.**

|  | **Treatment** | **This study (original analysis)** | **This study (De Smet et al 2016's approaches)** | **Increase using De Smet approaches** |
| --- | --- | --- | --- | --- |
| **Endometabolome** | **Cyanovirocells** | 0 | 17 | 17× |
|  | **Cyanovirocells with Protist** | 10 | 52 | 5× |
| **Exometabolome** | **Cyanovirocells** | 23 | 59 | 3× |
|  | **Cyanovirocells with Protist** | 15 | 38 | 3× |

**Supplementary Table S4: Phage S-SSM5 non-structural and non-lysis genes with known functions that are expressed during infection.** Metabolism code: Amino acid metabolism (A), Carbon & energy metabolisms (C), DNA metabolism (D) & nucleotide metabolism (D/N), Gene expression (E), Global metabolisms & stress response (G), Iron metabolism (I), Lysis (L), Photosynthesis (P), Phosphate stress (S), Virion/structural (V).

| Gene | **Final function** | Metabolism | Strand | Start (bp) | Stop(bp) |
| --- | --- | --- | --- | --- | --- |
| SSSM5_041 | **carbamoyltransferase/Putative Syn9_structural** | A | - | 32513 | 34177 |
| SSSM5_003 | **Hypothetical protein/Putative Syn9_transaldolase (TalC)** | C | + | 1022 | 1138 |
| SSSM5_017 | **Calvin cycle inhibitor CP12/Putative Syn9_gp17_CP12** | C | + | 14127 | 14354 |
| SSSM5_045 | **Hypothetical protein/Putative Syn9_transaldolase (TalC)** | C | + | 35734 | 36078 |
| SSSM5_114 | **6-phosphogluconate dehydrogenase (Gnd)** | C | + | 103077 | 104117 |
| SSSM5_116 | **glucose 6-phosphate dehydrogenase (Zwf)** | C | + | 104338 | 105780 |
| SSSM5_137 | **methylamine utilization protein** | C | + | 120873 | 121304 |
| SSSM5_195 | **Transaldolase (TalC)** | C | + | 160088 | 160768 |
| SSSM5_001 | **loader of T4 gp41 helicase** | D | + | 1 | 600 |
| SSSM5_004 | **ssDNA binding protein** | D | + | 1081 | 2061 |
| SSSM5_005 | **Hypothetical protein/Putative Syn9_2OG-Fe(II) oxygenase (DNA repair?)** | D | + | 2123 | 2293 |
| SSSM5_021 | **Hypothetical protein/Putative Syn9_DNA polymerase** | D | + | 15401 | 15568 |
| SSSM5_030 | **Hypothetical protein/Putative Syn9_endonuclease** | D | + | 20299 | 20655 |
| SSSM5_031 | **phosphoribosylaminoimidazole synthetase (purM) /Putative Syn9_structural** | D | + | 20655 | 21335 |
| SSSM5_059 | **Hypothetical protein/Putative Syn9_ribonucleotide reductase beta (NrdB)** | D | + | 40412 | 40912 |
| SSSM5_061 | **helicase** | D | + | 42207 | 43442 |
| SSSM5_062 | **DNA adenine methylase (Dam) [R/M]** | D | + | 43442 | 44299 |
| SSSM5_071 | **Hypothetical protein/Putative Syn9_DNA metabolism** | D | + | 46321 | 46539 |
| SSSM5_072 | **Hypothetical protein/Putaive Syn9_DNA metabolism** | D | + | 46540 | 46782 |
| SSSM5_118 | **Hypothetical protein/Putative Syn9_endonuclease** | D | + | 106058 | 106525 |
| SSSM5_134 | **DNA repair/recombination UvsY** | D | + | 118454 | 118882 |
| SSSM5_136 | **RNA-DNA and DNA-DNA helicase/Putative Syn9_UvsW helicase** | D | + | 119408 | 120880 |
| SSSM5_139 | **recombination endonuclease subunit 1** | D | + | 121864 | 122898 |
| SSSM5_141 | **recombination endonuclease subunit 2** | D | + | 123155 | 124876 |
| SSSM5_145 | **putative cobalt chelatase subunit CobS/Putative Syn9_153** | D/N | + | 127495 | 128568 |
| SSSM5_147 | **sliding clamp DNA polymerase accessory protein** | D | + | 128846 | 129505 |
| SSSM5_149 | **clamp loader subunit 1** | D | + | 129838 | 130782 |
| SSSM5_150 | **DNA endonuclease V/Putative Syn9_gp23** | D | + | 130779 | 131309 |
| SSSM5_153 | **Cytosine-specific DNA methyltransferase** | D | + | 131742 | 132575 |
| SSSM5_154 | **clamp loader subunit 2** | D | + | 132565 | 132966 |
| SSSM5_157 | **plasmid stability protein** | D | + | 133872 | 134114 |
| SSSM5_168 | **DNA polymerase (T4 g43)** | D | + | 139996 | 142494 |
| SSSM5_170 | **RecA recombination protein UvsX** | D | + | 142643 | 143653 |
| SSSM5_172 | **DNA primase-helicase (T4 g61)** | D | + | 144206 | 145585 |
| SSSM5_179 | **gp185** | D | + | 150078 | 151667 |
| SSSM5_188 | **DNA primase (T4 g61)** | D | + | 154945 | 155685 |
| SSSM5_189 | **ribonucleoside-diphosphate reductase subunit (NrdA)** | D/N | + | 155670 | 157967 |
| SSSM5_190 | **ribonucleotide reductase subunit B (NrdB)** | D/N | + | 157948 | 159114 |
| SSSM5_198 | **NAD synthetase/Putative Syn9_gp202** | D | + | 161059 | 161544 |
| SSSM5_206 | **Hypothetical protein/Putative Syn9_DNA metabolism** | D | + | 163003 | 163260 |
| SSSM5_218 | **glutaredoxin (NrdC)** | D/N | + | 170539 | 170781 |
| SSSM5_224 | **thymidylate synthase (Td)** | D/N | + | 172399 | 173100 |
| SSSM5_227 | **exonuclease/Putative Syn9_gp232** | D | + | 174098 | 174775 |
| SSSM5_020 | **tRNA-Val** | E | + | 15314 | 15385 |
| SSSM5_027 | **tRNA-Leu** | E | + | 19383 | 19467 |
| SSSM5_028 | **tRNA-Thr** | E | + | 19470 | 19543 |
| SSSM5_138 | **RNA polymerase sigma factor for late transcription** | E | + | 121397 | 121867 |
| SSSM5_155 | **translational repressor** | E | + | 132963 | 133391 |
| SSSM5_200 | **tRNA-Arg** | E | + | 161700 | 161772 |
| SSSM5_228 | **late promoter transcription accessory protein** | E | + | 174714 | 175019 |
| SSSM5_006 | **cAMP phosphodiesterase/Putative Syn9_glucose 6-P dehydrogenase (Zwf)** | G | + | 2290 | 2715 |
| SSSM5_018 | **2OG-Fe(II) oxygenase** | G | + | 14344 | 14880 |
| SSSM5_029 | **2OG-Fe(II) oxygenase** | G | + | 19680 | 20282 |
| SSSM5_042 | **2OG-Fe(II) oxygenase** | G | + | 34202 | 34759 |
| SSSM5_046 | **TPR domain/Putative Syn9_gp6** | G | + | 36080 | 36610 |
| SSSM5_048 | **leprecan-like 1 isoform 2/Putative Syn9_2OG-Fe(II) oxygenase** | G | + | 37138 | 37656 |
| SSSM5_135 | **2OG-Fe(II) oxygenase** | G | + | 118882 | 119427 |
| SSSM5_142 | **Rnf-Nqr/Putative Syn9_gp149** | G | + | 124845 | 125078 |
| SSSM5_163 | **Heat shock protein (Hsp20)** | G | + | 137271 | 137726 |
| SSSM5_171 | **2OG-Fe(II) oxygenase** | G | + | 143643 | 144209 |
| SSSM5_173 | **Pyrophosphatase/Putative Syn9_Nucleotide pyrophosphohydrolase (MazG)** | G | + | 145578 | 145979 |
| SSSM5_178 | **Cytidyltransferase/Putative Syn9_gp184** | G | + | 148916 | 150076 |
| SSSM5_229 | **tryptophan halogenase (antibiotic synthesis)** | G | + | 175016 | 176182 |
| SSSM5_043 | **ferrochelatase/Putative Syn9_gp54 /Fe transporter** | I | + | 34732 | 35322 |
| SSSM5_019 | **antenna protein/Putaive Syn9_CepT** | P | + | 14864 | 15322 |
| SSSM5_111 | **Plastocyanin (PetE)** | P | + | 102087 | 102410 |
| SSSM5_112 | **plastoquinol terminal oxidase (PTOX)** | P | + | 102407 | 102910 |
| SSSM5_148 | **Polyamine biosynthesis SpeD/Putative Syn9_prohead core protein** | P | + | 129507 | 129848 |
| SSSM5_204 | **High light inducible protein 1 (Hli)** | P | + | 162722 | 162940 |
| SSSM5_205 | **High light inducible protein 2 (Hli)** | P | + | 162974 | 163081 |
| SSSM5_211 | **photosystem II D1 protein (PsbA)** | P | + | 164719 | 165795 |
| SSSM5_213 | **photosystem II D2 protein (PsbD)** | P | + | 166122 | 167177 |
| SSSM5_165 | **Phosphate transporter subunit (PstS)** | S | + | 138186 | 139166 |
| SSSM5_226 | **phosphate-starvation inducible protein (PhoH)** | S | + | 173337 | 174098 |

**Supplementary Table S5: *Synechococcus* differentially expressed genes (DE) involved in the metabolisms reprogrammed by phage infection.**

| **ID** | **Acronym** | **Function** | **Metabolism** | **Genomic island** | **DE with phage only** | **DE with phage+protist** |
| --- | --- | --- | --- | --- | --- | --- |
| TX72_RS10955 | hli | high light inducible protein | Photosynthesis |  | No | Yes |
| TX72_RS12145 | hli | high light inducible protein | Photosynthesis |  | No | Yes |
| TX72_RS04020 | hli | high light inducible protein | Photosynthesis |  | No | Yes |
| TX72_RS07290 | hli | high light inducible protein | Photosynthesis | ISL9 | Yes | Yes |
| TX72_RS06895 | cpeT | carotenoid binding protein | Photosynthesis | ISL9 | No | Yes |
| TX72_RS06095 | pcxA | proton extrusion protein PcxA | Photosynthesis |  | No | Yes |
| TX72_RS10105 | cpeC | phycoerythrin class 2 subunit gamma linker polypeptide | Photosynthesis | PBS | No | Yes |
| TX72_RS11185 | ndhO | NADPH quinone oxidoreductase subunit O | Photosynthesis |  | No | Yes |
| TX72_RS09790 | cydB | cytochrome D ubiquinol oxidase subunit II | Photosynthesis | ISL12 | No | Yes |
| TX72_RS06400 | petF | ferredoxin petF | Photosynthesis |  | No | Yes |
| TX72_RS06770 | petF | ferredoxin petF | Photosynthesis | ISL9 | No | Yes |
| TX72_RS11290 | ycf3 | photosystem I assembly protein Ycf3 | Photosynthesis |  | No | Yes |
| TX72_RS09135 | pstS | phosphate ABC transporter substrate binding protein PstS | Phosphate stress response |  | Yes | Yes |
| TX72_RS00605 | phoA | alkaline phosphatase | Phosphate stress response |  | No | Yes |
| TX72_RS11545 | phoB | DNA binding response regulator (PhoB) | Phosphate stress response |  | No | Yes |
| TX72_RS09785 | phoH | phoH family protein | Phosphate stress response | ISL12 | Yes | Yes |
| TX72_RS11530 | nagA | N acetylglucosamine 6 phosphate deacetylase | Central C metabolism |  | Yes | No |
| TX72_RS00740 | cpsB | mannose 1 phosphate guanylyltransferase mannose 6 phosphate isomerase | Central C metabolism |  | Yes | Yes |
| TX72_RS03880 | fbaA | fructose 1 6 bisphosphate aldolase | Glycolysis/Calvin cycle |  | No | Yes |
| TX72_RS08865 | talB | transaldolase | Calvin cycle |  | No | Yes |
| TX72_RS08660 | rbcL | ribulose bisphosphate carboxylase large chain | Calvin cycle |  | No | Yes |
| TX72_RS08655 | rbcS | ribulose bisphosphate carboxylase small subunit | Calvin cycle |  | No | Yes |
| TX72_RS04810 | prs | ribose phosphate pyrophosphokinase | Nucleotide metabolism |  | No | Yes |
| TX72_RS11915 | ndk | nucleoside diphosphate kinase | Nucleotide metabolism |  | No | Yes |

**Supplementary Table S6: Metabolites significantly changing in the cyanovirocells with or without the protist.** Blank cells denote metabolites that were not significantly changing.

|  |  | **Changing with Protist** | | **Changing with Phage** | | **Changing with Protist + Phage** | |
| --- | --- | --- | --- | --- | --- | --- | --- |
| **Metabolite** | **Category** | **Endometabolite** | **Exometabolite** | **Endometabolite** | **Exometabolite** | **Endometabolite** | **Exometabolite** |
| 2-Deoxycytidine | Nucleotide derivatives |  |  |  | Yes |  | Yes |
| 3-methyladenine | Nucleotide derivatives |  |  |  | Yes |  |  |
| 5-Cytidylic acid | Nucleotide derivatives |  |  |  |  |  | Yes |
| 5-Hydroxymethyluracil | Nucleotide derivatives |  |  |  | Yes |  |  |
| Adenine | Nucleotide derivatives |  |  |  | Yes |  | Yes |
| Adenosine | Nucleotide derivatives |  |  |  | Yes |  | Yes |
| Cytosine | Nucleotide derivatives |  |  |  | Yes |  | Yes |
| Dihydrouracil | Nucleotide derivatives |  |  |  | Yes |  |  |
| Guanine | Nucleotide derivatives |  |  |  | Yes | Yes | Yes |
| Guanosine | Nucleotide derivatives |  |  |  |  |  | Yes |
| Hypoxanthine | Nucleotide derivatives |  |  |  | Yes |  |  |
| Xanthine | Nucleotide derivatives |  |  |  | Yes |  |  |
| 2-Methylglutaric acid | Amino acid derivatives |  |  |  |  |  | Yes |
| 3-(2-Hydroxyphenyl)propanoic acid | Amino acid derivatives |  |  |  | Yes |  |  |
| 4-hydroxyphenylacetic acid | Amino acid derivatives |  |  |  | Yes |  | Yes |
| 4-Methyl-2-oxovaleric acid | Amino acid derivatives |  |  |  |  |  | Yes |
| Cinnamic acid | Amino acid derivatives |  | Yes |  |  |  |  |
| Creatinine | Amino acid derivatives |  |  |  | Yes |  |  |
| DL-Leucine | Amino acid derivatives |  |  |  | Yes |  |  |
| L-glutamate | Amino acid derivatives |  |  |  | Yes |  |  |
| L-homoserine | Amino acid derivatives |  |  |  |  | Yes |  |
| L-Pipecolic acid | Amino acid derivatives |  |  |  | Yes |  |  |
| Methylthioadenosine | Amino acid derivatives |  |  |  | Yes |  |  |
| Biotin | Vitamins |  |  |  | Yes |  |  |
| Lumazine | Vitamins |  |  |  | Yes |  | Yes |
| Nicotinamide | Vitamins |  |  |  | Yes | Yes |  |
| 2-Aminobenzenesulfonic acid | Source of energy |  |  |  | Yes |  |  |
| Isocitric acid | Source of energy |  |  |  |  |  | Yes |
| L-Carnitine | Source of energy/stress |  |  |  | Yes |  | Yes |
| Pyrocatechol | Source of energy |  | Yes |  |  |  |  |
| Tetradecanoic acid | Lipid |  |  |  |  | Yes |  |
| Phenylacetic acid | Communication molecule |  |  |  | Yes |  | Yes |
| Trimethyl glycine | Stress molecule |  |  |  |  |  | Yes |
| Diethanolamine | Source of energy |  |  |  |  | Yes |  |
| unk129 2pt69 | Other |  |  |  |  | Yes |  |
| unk138p0550 | Other |  |  |  |  | Yes |  |
| unk143 1pt62 | Other |  |  |  |  | Yes |  |
| unk150 8pt19 | Other |  |  |  |  | Yes |  |
| unk241 12pt11 | Other |  |  |  |  | Yes |  |

**SUPPLEMENTARY FIGURE LEGENDS**

From figure 4. **Abbreviations for the compounds**: G6P:Glucose-6-phosphate; 6PGL: 6-phosphate gluconolactone; 6PG: 6-phosphate gluconate; Ru5P: ribulose-5-phosphate; RuBP: ribulose 1,5-bis-phosphate; PGA: 3-phosphoglycerate; BPGA: 1,3-bisphosphoglycerate; GAP: glyceraldehyde-3-phosphate; 3-C-1-Mpyr: 3-Carboxy-1-methylpyridinium; FBP: fructose-1,6-bisphosphate; F6P: fructose-6-phosphate; PRPP: 5-phospho-D-ribose alpha-1-pyrophosphate; F6P: fructose-6-phosphate; M6P: mannose-6-phosphate; GDP-M: GDP-Mannose; N-G6P: N-acetyl-galactosamine-6-phosphate; R5P: ribose-5-phosphate; IMP: inosinic acid; IDP: inosine diphosphate; ITP: inosine triphosphate; GTP: Guanosine triphosphate; dGTP: Deoxyguanosine triphosphate; DGP: Guanosine diphosphate; dGDP: Deoxyguanosine diphosphate; GMP: Guanosine monophosphate; ADP: Adenosine diphosphate; dADP: Deoxyadenosine diphosphate; ATP: Adenosine triphosphate; dATP: Deoxyadenosine triphosphate; CTP: Cytidine triphosphate; CDP: Cytidine diphosphate; CMP: Cytidine monophosphate; dCDP: Deoxycitidine diphosphate; dCTP: Deoxycitidine triphosphate; dCMP: Deoxycitidine monophosphate; UMP: Uridine monophosphate; UTP: Uridine triphosphate; dUTP: Deoxyuridine triphosphate; dUDP: Deoxyuridine diphosphate; dUMP: Deoxyuridine monophosphate; UDP: Uridine diphosphate; dTTP: Deoxythymidine triphosphate; dTDP: Deoxythymidine diphosphate; dTMP: Deoxythymidine monophosphate. **Abbreviations for the genes and proteins:** *psbAD*: photosystem II protein D1/D2; *speD*: polyamine biosynthesis; *hli*: high light inducible; *pcxA*: proton exclusion protein; *cpeC*: phycobilisome; *cpeT*: antenna protein; *ptox*: plastid terminal oxidase; *ndhO*: NADPH quinone oxidoreductase subunit O; *cydB*: cytochrome D ubiquinol oxidase subunit II; *petE*: plastocyanin; *petF*: ferredoxin; *ycf3*: photosystem I assembly protein; PSI/II: photosystem I/II; NDH: NAD(P)H dehydrogenase-like complex; *pstS:* phosphate ABC transporter periplasmic binding protein; *pstA*: phosphate ABC transporter membrane subunit; *pstB*: phosphate ABC transporter ATP-binding subunit; *pstC*: phosphate ABC transporter membrane subunit PstC; *phoA*: alkaline phosphatase; *phoH*: ATP-binding protein; *phoB*: DNA-binding transcriptional regulator; *cpsB*: mannose 1 phosphate guanylyltransferase mannose 6 phosphate isomerase; *fbaA*: fructose-1,6-bisphosphate aldolase; *nagA*: N-acetylglucosamine-6-phosphate deacetylase; *zwf*: glucose-6-phosphate dehydrogenase; *gnd*: 6-phosphogluconate dehydrogenase; *talBC:* transaldolase; *rbcLS*: rubisco; *cp12*: calvin cycle inhibitor protein cp12; *prs*: ribose phosphate pyrophosphokinase; *ndk*: nucleoside diphosphate kinase; *nrdABC*: ribonucleotide reductase; *cobS*: cobalt chelatase; *td*: thymidylate synthase.

**SUPPLEMENTARY REFERENCES**

1. Brussaard CPD. Optimization of procedures for counting viruses by flow cytometry. Appl Environ Microbiol. 2004 Mar;70(3):1506–13.

2. Hoff JC, Jakubowski W. Application of an end point dilution method to bacteriophage assay. Appl Microbiol. 1966;14(3):468–9.

3. Jarvis B, Wilrich C, Wilrich P-T. Reconsideration of the derivation of Most Probable Numbers, their standard deviations, confidence bounds and rarity values. J Appl Microbiol. 2010 Nov;109(5):1660–7.

4. Evans GT. The encounter speed of moving predator and prey. J Plankton Res [Internet]. 1989 Jan 1;11(2):415–7. Available from: https://doi.org/10.1093/plankt/11.2.415

5. Cosson J, Cachon M, Cachon J, Cosson MP. Swimming behaviour of the unicellular biflagellate Oxyrrhis marina: in vivo and in vitro movement of the two flagella. Biol cell. 1988;63(2):117–26.

6. Brahamsha B. An abundant cell-surface polypeptide is required for swimming by the nonflagellated marine cyanobacterium Synechococcus. Proc Natl Acad Sci U S A [Internet]. 1996 Jun 25;93(13):6504–9. Available from: https://pubmed.ncbi.nlm.nih.gov/8692845

7. Kiørboe T. A Mechanistic Approach to Plankton Ecology [Internet]. Princeton University Press; 2008. Available from: https://books.google.com/books?id=H6fLBGvKg6AC

8. Boakes DE, Codling EA, Thorn GJ, Steinke M. Analysis and modelling of swimming behaviour in Oxyrrhis marina. J Plankton Res [Internet]. 2011 Apr 1;33(4):641–9. Available from: https://doi.org/10.1093/plankt/fbq136

9. Montagnes DJS, Lowe CD, Roberts EC, Breckels MN, Boakes DE, Davidson K, et al. An introduction to the special issue: Oxyrrhis marina, a model organism? J Plankton Res [Internet]. 2011 Apr 1;33(4):549–54. Available from: https://doi.org/10.1093/plankt/fbq121

10. Brew HS, Moran SB, Lomas MW, Burd AB. Plankton community composition, organic carbon and thorium-234 particle size distributions, and particle export in the Sargasso sea. J Mar Res. 2009;67(6):845–68.

11. Johnson MD, Rome M, Stoecker DK. Microzooplankton grazing on Prorocentrum minimum and Karlodinium micrum in Chesapeake Bay. Limnol Oceanogr [Internet]. 2003 Jan 1;48(1):238–48. Available from: https://doi.org/10.4319/lo.2003.48.1.0238

12. Duhamel S, Kim E, Sprung B, Anderson OR. Small pigmented eukaryotes play a major role in carbon cycling in the P-depleted western subtropical North Atlantic, which may be supported by mixotrophy. Limnol Oceanogr [Internet]. 2019 Nov 1;64(6):2424–40. Available from: https://doi.org/10.1002/lno.11193

13. Ohio Supercomputer Center [Internet]. 1987. Available from: http://osc.edu/ark:/19495/f5s1ph73

14. Robinson MD, McCarthy DJ, Smyth GK. edgeR: a Bioconductor package for differential expression analysis of digital gene expression data. Bioinformatics [Internet]. 2009/11/17. 2010;26(1):139–40. Available from: http://www.ncbi.nlm.nih.gov/pubmed/19910308

15. Mortazavi A, Williams BA, McCue K, Schaeffer L, Wold B. Mapping and quantifying mammalian transcriptomes by RNA-Seq. Nat Methods [Internet]. 2008;5(7):621–8. Available from: http://www.ncbi.nlm.nih.gov/pubmed/18516045

16. Wickham H. ggplot2: Elegant Graphics for Data Analysis [Internet]. Springer-Verlag New York; 2016. Available from: https://ggplot2.tidyverse.org

17. Smolkin M, Ghosh D. Cluster stability scores for microarray data in cancer studies. BMC Bioinformatics. 2003/09/10. 2003;4:36.

18. Keseler IM, Mackie A, Santos-Zavaleta A, Billington R, Bonavides-Martínez C, Caspi R, et al. The EcoCyc database: reflecting new knowledge about Escherichia coli K-12. Nucleic Acids Res [Internet]. 2016;45(D1):D543–50. Available from: https://doi.org/10.1093/nar/gkw1003

19. Yao Y, Sun T, Wang T, Ruebel O, Northen T, Bowen BP. Analysis of Metabolomics Datasets with High-Performance Computing and Metabolite Atlases. Metabolites [Internet]. 2015 Jul 20;5(3):431–42. Available from: https://pubmed.ncbi.nlm.nih.gov/26287255

20. Steenwyk JL, Rokas A, Newton ILG. ggpubfigs: Colorblind-Friendly Color Palettes and ggplot2 Graphic System Extensions for Publication-Quality Scientific Figures. Microbiol Resour Announc. 2021;10(44):e00871-21.

21. De Smet J, Zimmermann M, Kogadeeva M, Ceyssens P-J, Vermaelen W, Blasdel B, et al. High coverage metabolomics analysis reveals phage-specific alterations to Pseudomonas aeruginosa physiology during infection. ISME J [Internet]. 2016 Aug;10(8):1823–35. Available from: https://www.nature.com/articles/ismej20163

22. Apple JK, Strom SL, Palenik B, Brahamsha B. Variability in protist grazing and growth on different marine Synechococcus isolates. Appl Environ Microbiol [Internet]. 2011/03/11. 2011 May;77(9):3074–84. Available from: https://pubmed.ncbi.nlm.nih.gov/21398485

23. Straile D. Gross growth efficiencies of protozoan and metazoan zooplankton and their dependence on food concentration, predator-prey weight ratio, and taxonomic group. Limnol Oceanogr [Internet]. 1997 Sep 1;42(6):1375–85. Available from: https://doi.org/10.4319/lo.1997.42.6.1375

24. Hammer A, Grüttner C, Schumann R. The Effect of Electrostatic Charge of Food Particles on Capture Efficiency by Oxyrrhis marina Dujardin (Dinoflagellate). Protist [Internet]. 1999;150(4):375–82. Available from: https://www.sciencedirect.com/science/article/pii/S1434461099700398

25. Avrani S, Wurtzel O, Sharon I, Sorek R, Lindell D. Genomic island variability facilitates Prochlorococcus-virus coexistence. Nature. 2011/07/02. 2011;474(7353):604–8.

26. Fernández L, Rodríguez A, García P. Phage or foe: an insight into the impact of viral predation on microbial communities. ISME J [Internet]. 2018; Available from: https://doi.org/10.1038/s41396-018-0049-5

27. Roberts EC, Wootton EC, Davidson K, Jeong HJ, Lowe CD, Montagnes DJS. Feeding in the dinoflagellate Oxyrrhis marina: Linking behaviour with mechanisms. J Plankton Res. 2011;33(4):603–14.

28. Guo Z, Zhang H, Liu S, Lin S. Biology of the Marine Heterotrophic Dinoflagellate Oxyrrhis marina: Current Status and Future Directions. Microorganisms [Internet]. 2013 Oct 21;1(1):33–57. Available from: https://pubmed.ncbi.nlm.nih.gov/27694763

29. Harke MJ, Jankowiak JG, Morrell BK, Gobler CJ. Transcriptomic Responses in the Bloom-Forming Cyanobacterium &lt;span class=&quot;named-content genus-species&quot; id=&quot;named-content-1&quot;&gt;Microcystis&lt;/span&gt; Induced during Exposure to Zooplankton. Stabb E V, editor. Appl Environ Microbiol [Internet]. 2017 Mar 1;83(5):e02832-16. Available from: http://aem.asm.org/content/83/5/e02832-16.abstract

30. Beisser D, Bock C, Hahn MW, Vos M, Sures B, Rahmann S, et al. Interaction-specific changes in the transcriptome of polynucleobacter asymbioticuscaused by varying protistan communities. Front Microbiol [Internet]. 2019;10(JULY):1–14. Available from: https://www.frontiersin.org/articles/10.3389/fmicb.2019.01498/full#h3

31. Fenchel T, Blackburn N. Motile Chemosensory Behaviour of Phagotrophic Protists: Mechanisms for and Efficiency in Congregating at Food Patches. Protist [Internet]. 1999;150(3):325–36. Available from: https://www.sciencedirect.com/science/article/pii/S1434461099700337

32. Wolfe G V. The chemical defense ecology of marine unicellular plankton: constraints, mechanisms, and impacts. Biol Bull. 2000 Apr;198(2):225–44.

33. Long JD, Smalley GW, Barsby T, Anderson JT, Hay ME. Chemical cues induce consumer-specific defenses in a bloom-forming marine phytoplankton. Proc Natl Acad Sci [Internet]. 2007 Jun 19;104(25):10512 LP – 10517. Available from: http://www.pnas.org/content/104/25/10512.abstract

34. Selander E, Jakobsen HH, Lombard F, Kiørboe T. Grazer cues induce stealth behavior in marine dinoflagellates. Proc Natl Acad Sci [Internet]. 2011 Mar 8;108(10):4030 LP – 4034. Available from: http://www.pnas.org/content/108/10/4030.abstract

35. Selander E, Thor P, Toth G, Pavia H. Copepods induce paralytic shellfish toxin production in marine dinoflagellates. Proceedings Biol Sci [Internet]. 2006 Jul 7;273(1594):1673–80. Available from: https://pubmed.ncbi.nlm.nih.gov/16769640

36. Harvey EL, Menden-Deuer S. Predator-Induced Fleeing Behaviors in Phytoplankton: A New Mechanism for Harmful Algal Bloom Formation? PLoS One [Internet]. 2012 Sep 28;7(9):e46438. Available from: https://doi.org/10.1371/journal.pone.0046438

37. Harvey EL, Jeong HJ, Menden-Deuer S. Avoidance and attraction: Chemical cues influence predator-prey interactions of planktonic protists. Limnol Oceanogr [Internet]. 2013 Jul 1;58(4):1176–84. Available from: https://doi.org/10.4319/lo.2013.58.4.1176

38. Bailly-Bechet M, Vergassola M, Rocha E. Causes for the intriguing presence of tRNAs in phages. Genome Res [Internet]. 2007;17(10):1486–95. Available from: http://www.ncbi.nlm.nih.gov/pubmed/17785533

39. Limor-Waisberg K, Carmi A, Scherz A, Pilpel Y, Furman I. Specialization versus adaptation: two strategies employed by cyanophages to enhance their translation efficiencies. Nucleic Acids Res [Internet]. 2011/04/05. 2011 Aug;39(14):6016–28. Available from: https://www.ncbi.nlm.nih.gov/pubmed/21470965

40. Dailey HA, Dailey TA, Gerdes S, Jahn D, Jahn M, O&#039;Brian MR, et al. Prokaryotic Heme Biosynthesis: Multiple Pathways to a Common Essential Product. Microbiol Mol Biol Rev [Internet]. 2017 Mar 1;81(1):e00048-16. Available from: http://mmbr.asm.org/content/81/1/e00048-16.abstract

41. Rao VB, Black LW. Structure and assembly of bacteriophage T4 head. Virol J [Internet]. 2010;7(1):356. Available from: https://doi.org/10.1186/1743-422X-7-356

42. Sun S, Gao S, Kondabagil K, Xiang Y, Rossmann MG, Rao VB. Structure and function of the small terminase component of the DNA packaging machine in T4-like bacteriophages. Proc Natl Acad Sci U S A [Internet]. 2011/12/29. 2012 Jan 17;109(3):817–22. Available from: https://pubmed.ncbi.nlm.nih.gov/22207623

43. Doron S, Fedida A, Hernndez-Prieto MA, Sabehi G, Karunker I, Stazic D, et al. Transcriptome dynamics of a broad host-range cyanophage and its hosts. ISME J [Internet]. 2016;10(6):1437–55. Available from: http://dx.doi.org/10.1038/ismej.2015.210

44. Chung M, Bruno VM, Rasko DA, Cuomo CA, Muñoz JF, Livny J, et al. Best practices on the differential expression analysis of multi-species RNA-seq. Genome Biol [Internet]. 2021;22(1):121. Available from: https://doi.org/10.1186/s13059-021-02337-8

45. Zborowsky S, Lindell D. Resistance in marine cyanobacteria differs against specialist and generalist cyanophages. Proc Natl Acad Sci [Internet]. 2019; Available from: https://www.pnas.org/content/early/2019/08/02/1906897116

46. Howard-Varona C, Roux S, Dore H, Solonenko NE, Holmfeldt K, Markillie LM, et al. Regulation of infection efficiency in a globally abundant marine Bacteriodetes virus. ISME J [Internet]. 2017;11(1):284–95. Available from: http://www.nature.com/doifinder/10.1038/ismej.2016.81

47. Howard-Varona C, Hargreaves KR, Solonenko NE, Markillie LM, White RA, Brewer HM, et al. Multiple mechanisms drive phage infection efficiency in nearly identical hosts. ISME J [Internet]. 2018;12(6):1605–18. Available from: https://www.nature.com/articles/s41396-018-0099-8

48. Blasdel BG, Chevallereau A, Monot M, Lavigne R, Debarbieux L. Comparative transcriptomics analyses reveal the conservation of an ancestral infectious strategy in two bacteriophage genera. ISME J [Internet]. 2017 Sep;11(9):1988–96. Available from: https://www.ncbi.nlm.nih.gov/pubmed/28498372

49. Luke K, Radek A, Liu X, Campbell J, Uzan M, Haselkorn R, et al. Microarray analysis of gene expression during bacteriophage T4 infection. Virology [Internet]. 2002;299(2):182–91. Available from: http://www.ncbi.nlm.nih.gov/pubmed/12202221

50. Kutter E, Bryan D, Ray G, Brewster E, Blasdel B, Guttman B. From host to phage metabolism: Hot tales of phage T4’s takeover of E. coli [Internet]. Vol. 10, Viruses. 2018. Available from: https://www.ncbi.nlm.nih.gov/pubmed/30037085

51. Lin X, Ding H, Zeng Q. Transcriptomic response during phage infection of a marine cyanobacterium under phosphorus-limited conditions. Environ Microbiol. 2016;18(2):450–60.

52. Thompson LR, Zeng Q, Chisholm SW. Gene Expression Patterns during Light and Dark Infection of <italic>Prochlorococcus</italic> by Cyanophage. PLoS One [Internet]. 2016;11(10):e0165375. Available from: http://dx.doi.org/10.1371%2Fjournal.pone.0165375

53. Ankrah NYD, May AL, Middleton JL, Jones DR, Hadden MK, Gooding JR, et al. Phage infection of an environmentally relevant marine bacterium alters host metabolism and lysate composition. ISME J [Internet]. 2013 May;8(5):1089–100. Available from: http://www.ncbi.nlm.nih.gov/pubmed/24304672

54. Chevallereau A, Blasdel BG, De Smet J, Monot M, Zimmermann M, Kogadeeva M, et al. Next-Generation “-omics” Approaches Reveal a Massive Alteration of Host RNA Metabolism during Bacteriophage Infection of Pseudomonas aeruginosa. PLoS Genet [Internet]. 2016/07/06. 2016;12(7):e1006134. Available from: https://journals.plos.org/plosgenetics/article?id=10.1371/journal.pgen.1006134

55. Santos-Beneit F. The Pho regulon: a huge regulatory network in bacteria [Internet]. Vol. 6, Frontiers in Microbiology. 2015. p. 402. Available from: https://www.frontiersin.org/article/10.3389/fmicb.2015.00402

56. Friedman S, Gots JS. The purine and pyrimidine metabolism of normal and phage-infected Escherichia coli. J Biol Chem [Internet]. 1953 Mar;201(1):125–35. Available from: https://www.jbc.org/content/201/1/125.full.pdf

57. Zimmerman AE, Howard-Varona C, Needham DM, John SG, Worden AZ, Sullivan MB, et al. Metabolic and biogeochemical consequences of viral infection in aquatic ecosystems. Nat Rev Microbiol [Internet]. 2019; Available from: https://doi.org/10.1038/s41579-019-0270-x

58. Thompson LR, Zeng Q, Kelly L, Huang KH, Singer AU, Stubbe J, et al. Phage auxiliary metabolic genes and the redirection of cyanobacterial host carbon metabolism. Proc Natl Acad Sci U S A [Internet]. 2011;108(39):E757-64. Available from: http://www.ncbi.nlm.nih.gov/pubmed/21844365

59. Sharon I, Alperovitch A, Rohwer F, Haynes M, Glaser F, Atamna-Ismaeel N, et al. Photosystem I gene cassettes are present in marine virus genomes. Nature. 2009 Sep;461(7261):258–62.

60. Philosof A, Battchikova N, Aro E-M, Béjà O. Marine cyanophages: tinkering with the electron transport chain. ISME J [Internet]. 2011;5(10):1568–70. Available from: https://doi.org/10.1038/ismej.2011.43

61. Lindell D, Jaffe JD, Johnson ZI, Church GM, Chisholm SW. Photosynthesis genes in marine viruses yield proteins during host infection. Nature [Internet]. 2005;438(7064):86–9. Available from: http://www.ncbi.nlm.nih.gov/pubmed/16222247

62. Puxty RJ, Millard AD, Evans DJ, Scanlan DJ. Shedding new light on viral photosynthesis. Photosynth Res. 2015 Oct;126(1):71–97.

63. Puxty RJ, Millard AD, Evans DJ, Scanlan DJ. Viruses Inhibit CO2 Fixation in the Most Abundant Phototrophs on Earth. Curr Biol [Internet]. 2016 Jun 20;26(12):1585–9. Available from: https://doi.org/10.1016/j.cub.2016.04.036

64. Suggett DJ, MacIntyre HL, Geider RJ. Evaluation of biophysical and optical determinations of light absorption by photosystem II in phytoplankton. Limnol Oceanogr Methods [Internet]. 2004 Oct 1;2(10):316–32. Available from: https://doi.org/10.4319/lom.2004.2.316

65. Kolber ZS, Prasil O, Falkowski PG. Measurements of variable chlorophyll fluorescence using fast repetition rate techniques: defining methodology and experimental protocols. Biochim Biophys Acta. 1998 Oct;1367(1–3):88–106.

66. Kromkamp JC, Forster RM. The use of variable fluorescence measurements in aquatic ecosystems: differences between multiple and single turnover measuring protocols and suggested terminology. Eur J Phycol [Internet]. 2003 May 1;38(2):103–12. Available from: https://doi.org/10.1080/0967026031000094094

67. Suggett DJ, Moore CM, Oxborough K, Geider RJ. Fast Repetition Rate (FRR) Chlorophyll a Fluorescence Induction Measurements. Chelsea Technol Gr [Internet]. 2006;53. Available from: http://www.psi.cz/ftp/publications/kautsky/FRRFmethodsManual.pdf%5Cnpapers://64c875cb-35e9-49cc-86a7-69f7bda703f6/Paper/p289

68. Suggett DJ. 2010_Book_ChlorophyllAFluorescenceInAqua.pdf. 2010.

69. Clokie MRJ, Millard AD, Mann NH. T4 genes in the marine ecosystem: studies of the T4-like cyanophages and their role in marine ecology. Virol J [Internet]. 2010 Oct 28;7:291. Available from: https://www.ncbi.nlm.nih.gov/pubmed/21029435

70. Stirbet A, Lazár D, Papageorgiou GC, Govindjee. Chapter 5 - Chlorophyll a Fluorescence in Cyanobacteria: Relation to Photosynthesis☆. In: Mishra AK, Tiwari DN, Rai AN, editors. Cyanobacteria [Internet]. Academic Press; 2019. p. 79–130. Available from: https://www.sciencedirect.com/science/article/pii/B9780128146675000052
